# Characterization of the antifungal functions of a WGA-Fc (IgG2a) fusion protein binding to cell wall chitin oligomers

**DOI:** 10.1038/s41598-017-12540-y

**Published:** 2017-09-22

**Authors:** Susie Coutinho Liedke, Daniel Zamith Miranda, Kamilla Xavier Gomes, Jorge Luis S. Gonçalves, Susana Frases, Joshua D. Nosanchuk, Marcio L. Rodrigues, Leonardo Nimrichter, José Mauro Peralta, Allan J. Guimarães

**Affiliations:** 1 Departamento de Imunologia, Instituto de Microbiologia Professor Paulo de Góes, Universidade Federal do Rio de Janeiro, RJ, Brazil; 2 Laboratório de Glicobiologia de Eucariotos, Instituto de Microbiologia Paulo de Góes, Universidade Federal do Rio de Janeiro, RJ, Brazil; 3 Departments of Medicine and Microbiology and Immunology, Albert Einstein College of Medicine, Bronx, NY, USA; 4 Departamento de Microbiologia e Parasitologia, Instituto Biomédico, Universidade Federal Fluminense, Rio de Janeiro, Brazil; 5 Laboratório de Ultraestrutura Celular Hertha Meyer, Instituto de Biofísica Carlos Chagas Filho, Universidade Federal do Rio de Janeiro, RJ, Brazil; 6 Laboratório de Biologia Celular de Leveduras Patogênicas, Instituto de Microbiologia Professor Paulo de Góes, Universidade Federal do Rio de Janeiro, RJ, Brazil; 7 Fundação Oswaldo Cruz – Fiocruz, Centro de Desenvolvimento Tecnológico em Saúde (CDTS), Rio de Janeiro, Brazil

## Abstract

The majority of therapeutic strategies for mycosis require the protracted administration of antifungals, which can result in significant toxicities and have unacceptable failure rates. Hence, there is an urgent need for the development of improved therapeutic approaches, and monoclonal antibody-based drugs are potentially a powerful alternative to standard antifungals. To develop a broad antibody-like reagent against mycosis, wheat germ agglutinin (WGA) was linked to the effector Fc region of murine IgG2a. The resultant WGA-Fc displayed high affinity to purified chitin and bound efficiently to fungal cell walls, co-localizing with chitin, in patterns ranging from circular (*Histoplasma capsulatum*) to punctate (*Cryptococcus neoformans*) to labeling at the bud sites (*Candida albicans* and *Saccharomyces cerevisiae*). WGA-Fc directly inhibited fungal growth in standard cultures. WGA-Fc opsonization increased fungal phagocytosis, as well augmented the antifungal functions by macrophages. Prophylactic administration of WGA-Fc fully protected mice against *H. capsulatum*, correlating with a reduction in lung, spleen and liver fungal burdens. Administration of WGA-Fc also dramatically diminished pulmonary inflammation. Hence, the opsonic activity of WGA-Fc effectively modulates fungal cell recognition and promotes the elimination of fungal pathogens. Therefore, we propose WGA-Fc as a potential “pan-fungal” therapeutic that should be further developed for use against invasive mycoses.

## Introduction

Since the 1970s, there has been a considerable increase (>200%) in the incidence of human fungal infections^1,2^, particularly in individuals with compromised immunity, i.e., those with HIV infection, neoplasias, leukemias, autoimmune diseases, extremes of age or undergoing solid organ transplants^1^.

Relatively few antifungal drugs are available, and their efficacy is unacceptably poor for invasive mycoses as mortality rates remain high (i.e. candidiasis (33.9%), aspergillosis (23.3%), histoplasmosis (20.0%) and cryptococcosis (12.7%))^1^. Additionally, fungal drug resistance has continued to emerge^3,4^, and there are fungal pathogens for which no effective therapeutic options are available^5–7^. Moreover, current antifungal drugs have diverse and, potentially severe side effects, which impairs patient adherence and compliance^8^. Hence, there is a significant need for the development of new antifungal alternatives, especially for treating multidrug-resistant fungi and, potentially, for reducing the duration of therapy^3,7^.

Innovative approaches to combat invasive mycoses have included immunotherapeutic strategies^9–11^, such as vaccination and passive immunization. However, as the majority of invasive fungal infections occur primarily in immunocompromised individuals, vaccination might not be an effective approach^7,12^. However, the administration of antibody-based therapies can promote effective host responses against pathogenic fungi in diverse patient populations^7,9^. A potential drawback of this approach is that an antibody is generally organism specific, and a more effective strategy would use a reagent that recognizes diverse species as well as all isolates within a species^6,13,14^.

Common potential antigen targets, which also are important fungal virulence factors, include proteins, such as histone 2B (H2B)^15^ and heat shock proteins (Hsps)^16^, polysaccharides^17^, such as β-glucans^11^ and chitin^18^, lipids, such as glucosylceramide^19^, and polymers, such as melanin^20^. In fact, we have previously demonstrated that monoclonal antibodies (mAbs) targeting conserved fungal antigens can be generated using a specific fungus and applied therapeutically against additional fungal species^6,21,22^. Examples are IgMs to a histone 2B and IgGs to an Hsp60^15,16,23–25^. Additional mAbs to “universal” antigens that have been shown to bind diverse fungi include 2G8, an IgG2b against β-1,3-glucan^26^; 6D2, an IgM against melanin^27^ and B11, an IgM against glucosylceramide on the fungal surface^28,29^.

Chitin, an N-acetylglucosamine polymer, is a key constituent of the fungal cell wall^20,30^ and it is common to all known fungi, which makes it an excellent target for new therapeutic strategies^6,13^. However, the generation of specific and strongly binding antibodies against carbohydrate epitopes has been complicated as fungal glycans are generally considered to be classic T-cell independent antigens that stimulate a short lived humoral response^31–33^. Based on these concepts, we hypothesized that the construction of an Fc-fusion protein containing the polysaccharide binding site of the WGA, a lectin with high affinity to N-acetylglucosamine residues and sialic acid^34^, with the effector Fc portion of immunoglobulins (CH2 and CH3 domains) would have the recognition property of these common fungal structures, and would thus potentially function as an effective diagnostic and therapeutic for fungal pathogens. Interactions of opsonized fungi to host effector cells primarily occurs through Fc receptors on the surface of phagocytes, such as macrophages, dendritic cells and neutrophils, which subsequently triggers the effector functions of these cells and activates other components of innate immunity, such as the complement system^17,35^. Hence, we utilized the murine IgG2a Fc in constructing our WGA lectin chimera, as it is the IgG subclass that has the highest efficiency for inducing host cell effector responses^17,35^ and activating the complement system^36,37^.

In this work, we demonstrate that the WGA-Fc fusion protein is expressed as a dimer in solution and binds to chitin as efficiently as the native WGA protein. This fusion protein is also able to efficiently recognize chitin oligomers on the surface of diverse pathogenic fungi. Regarding its effector functions, the WGA-Fc is able to increase phagocytosis by macrophages, as well as their antifungal functions. *In vitro*, these reagents are also able to inhibit fungal growth. Moreover, the fusion proteins are highly effective against *H. capsulatum* in *in vivo* models, validating their biological efficacy. Therefore, our findings strongly suggest that the WGA-Fc fusion should be considered as a potential immunobiological drug for protection against mycosis.

## Results

### WGA-Fc fusion protein construction

**Figure 1:**
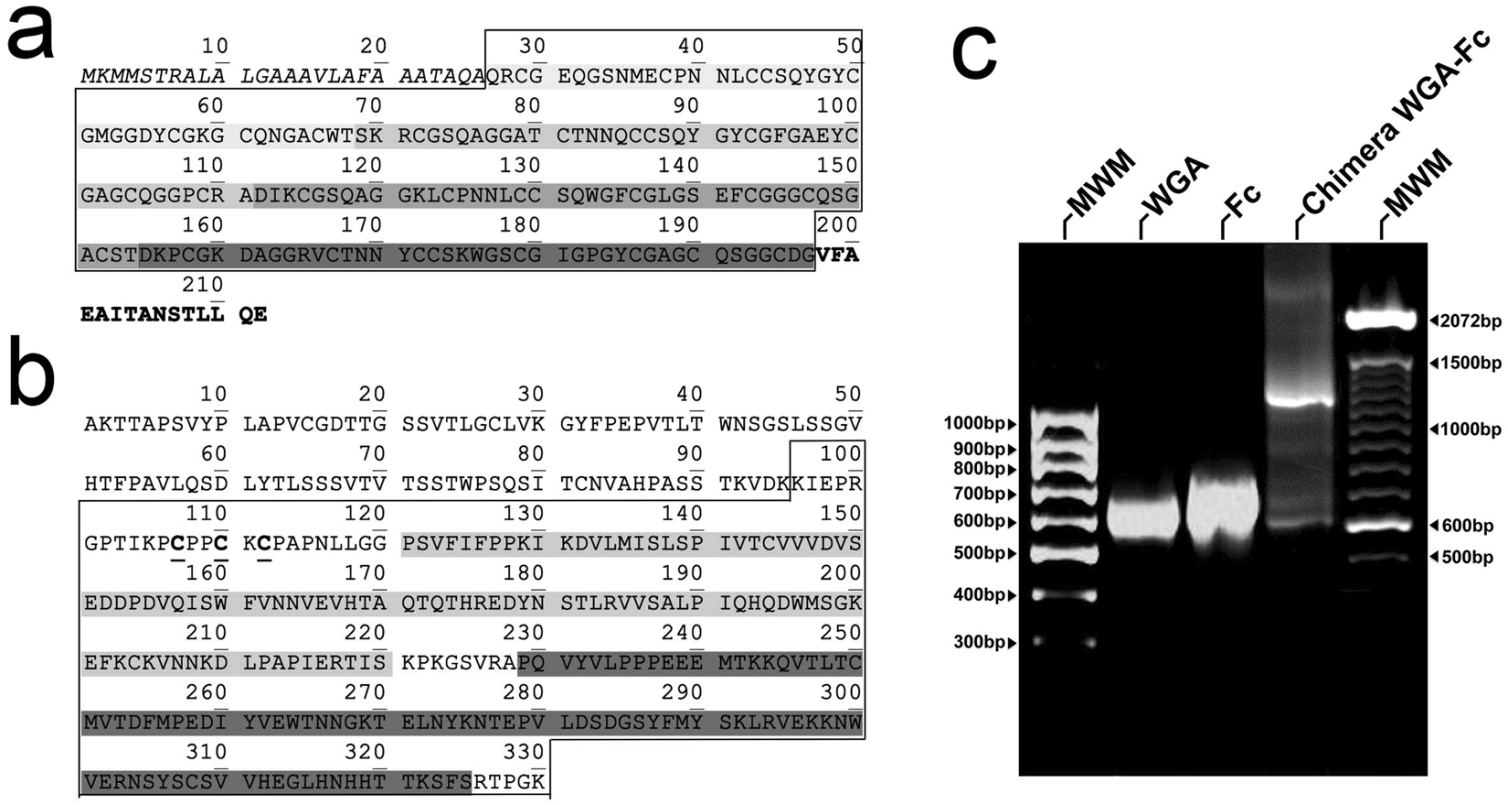
Domains considered for the production of the WGA-Fc chimera. (**a**) Amino acid sequence of WGA (GenBank AAA34256.1). The sequence within the polygon indicates the protein domain chosen for gene amplification of the WGA 1 cDNA. The sequences highlighted with varying grey tonalities (domains 1–4) display distinct protein domains that bind chitin oligomers (**b**) Amino acid sequence and domains of the IgG2a heavy chain (GenBank AB097847). The sequence with the polygon was selected for codifying cDNA amplification. The light grey indicates the CH2 domain, whereas the dark grey indicates the CH3 domain of the heavy chain. (**c**) Electrophoresis profiles of the amplified regions of WGA (lane 2), Fc (CH2 and CH3 domains; lane 3) and the fused sequence WGA-Fc (lane 4). First and last lanes on gel represent two molecular weight markers, 100pb GeneRuler and 100pb DNA Ladder (Life Technologies), respectively.

A recombinant gene composed of the respective coding sequence of the isolectin domain present in WGA 2 lectin (Fig. 1a) and the murine IgG2a hinge and CH2-CH3 domains (Fig. 1b) was constructed. Sequences of WGA and CH2-CH3 were amplified by PCR using the respective primers listed in the methods section, and the fragments were 590 bp and 732 bp as expected, respectively (Fig. 1c). A successful fusion of the sequences by overlapping PCR to produce the WGA-Fc chimeric gene rendered a product with 1304 bp in length (Fig. 1c). The subcloning of the fused sequence into the TOPO vector was confirmed by PCR and after endonuclease digestion, the fragment was ligated into the pSecTag2A eukaryotic expression vector.

### Molecular characterization of the WGA-Fc chimera

**Figure 2:**
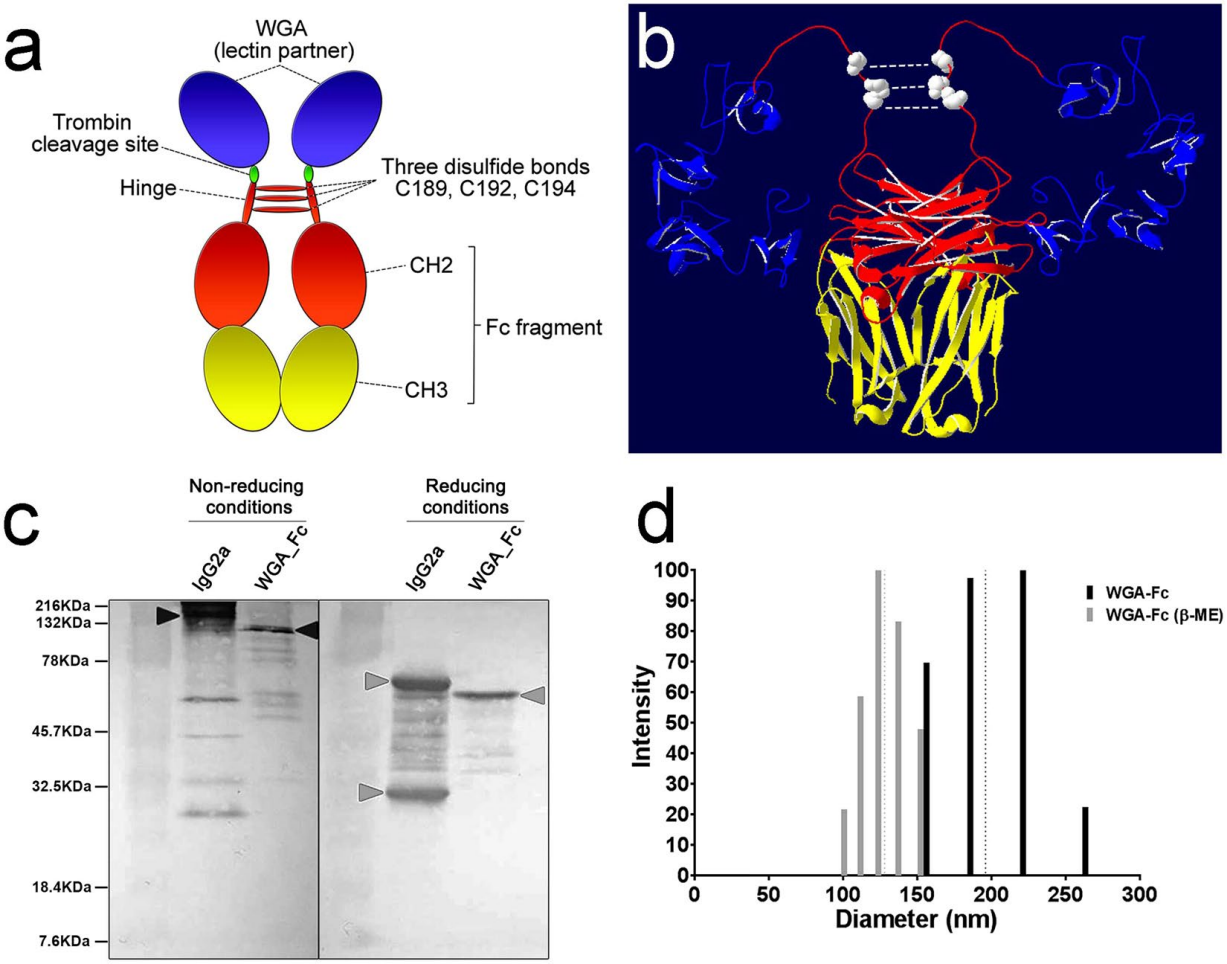
Structural schematics and characterization of the WGA-Fc. (**a**) A cartoon displaying the theoretical quaternary structure of the WGA-Fc chimera in solution upon association of two identical monomers, each indicated by the WGA domains (blue) fused to the CH2-CH3 regions (red and yellow), which are linked to each other by three disulfide bonds through cysteines at C189, C192 and C194. The CH2-CH3 domains form the Fc part of the chimera, WGA-Fc. (**b**) Molecular modeling of the WGA-Fc chimera, displaying the spatial arrangement of the 2 WGA domains (blue), a coiled domain bearing the C189, C192 and C194 cysteines (white dots) forming disulfide bond interchains, and the Fc fragment, structurally comprised by spatial arrangement of the CH2-CH3 domains of both chains. (**c**) Western blot using an anti-mouse IgG phosphatase conjugate for the detection of the WGA-Fc under non-reducing and reducing conditions. Under non-reducing conditions, both monomers are bound through the disulfide bonds, forming the WGA-Fc chimera with an approximate molecular weight of 97.5 kDa (as a control we can observe an IgG2a mAb with an approximate mass of 150 kDa, due to the association of 2 light and 2 heavy chains). Under reducing conditions, the quaternary structure of the double-chained WGA-Fc is broken into monomers, each approximately 48.7 kDa (as a control we can observe an IgG2a mAb with approximate 2 main bands of 25 and 53 kDa, which are respectively the light and heavy chains). (**d**) Dynamic light scattering displaying the reduction in WGA-Fc size (cystein bound double chain, dark bars) of approximately half of the dimension upon treatment under reducing conditions with β-mercaptoethanol (β-ME) and subsequent production of singlets (WGA-Fc’, grey bars). Dashed lines represent the effective diameter of WGA-Fc (dark) and WGA-Fc’ (grey) in solution.

We expected the WGA-Fc chimeras to form a dimer of 2 identical 412 aa chains in solution (171 aa from WGA + 6 aa from thrombin cleavage site +235 aa from IgG2a-Fc) (Fig. 2a) due to the presence of cysteines forming disulfide bonds on the positions 189, 192 and 194 of the newly fused chain (former cysteines 107, 110, 112 aa within the IgG2a heavy chain), resembling an antibody structure (Fig. 2b). Western blot analysis of the purified WGA-Fc chimeric protein using an anti-mouse IgG alkaline phosphatase conjugate under non-reducing conditions, revealed a 97.5-kDa product consistent with the double-chained quaternary structure of the WGA-Fc chimera, whereas the IgG2a control produced a 160-kDa product. Yet, under reducing conditions in the presence of β-mercaptoethanol, the IgG2a control displayed two bands with molecular masses of 25-kDa and 53-kDa corresponding to the light and heavy-chains of the 12D3 mAb, respectively. In contrast, the chimeric WGA-Fc protein displayed a 48.7-kDa product under reducing conditions, demonstrating that these monomers formed disulfide bonds (Fig. 2c). These results were confirmed by dynamic light scattering (DLS), which measured the hydrodynamic radius of the chimeras (Fig. 2d). In solution, the WGA-Fc hydrodynamic radius ranged from 156.2 to 263 nm, with an average radius of 196.1 nm. In comparison, the WGA-Fc, under denaturing condition in the presence of reducing agents, ranged from 100.8 to 152.4 nm, with an average radius of 128.0 nm (p < 0.05). This indicates that in solution, the WGA-Fc forms a dimer of 2 single chains, which could be observed as singlets after β-mercaptoethanol treatment.

### WGA-Fc binds to chitin oligomers similarly to native WGA

**Figure 3:**
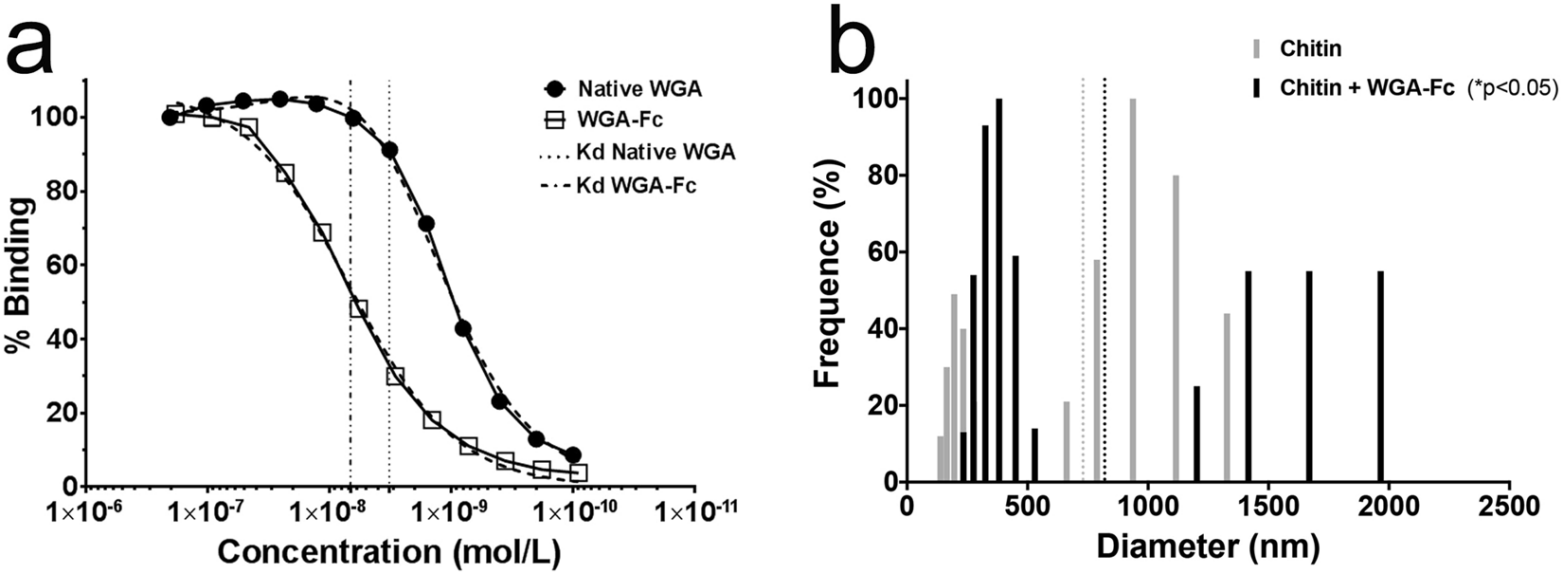
Characterization of WGA-Fc binding to purified chitin. (**a**) Indirect ELISA comparing the Kd (dissociation constants) of native WGA and WGA-Fc, within the same order of magnitude. (**b**) Dynamic light scattering of the chitin populations in solution (grey bars) and the increase in diameter upon incubation with WGA-Fc chimera (dark bars), indicating aggregation of chitin fibers. Dashed lines represent the effective diameter of chitin (grey) and chitin + WGA-Fc (dark) in solution.

WGA-Fc binding to chitin oligomers was examined and its dissociation constant compared to the native WGA. The native WGA effectively bound the chitin oligomers on the microplate, displaying a Kd of 3.2 × 10^−9^mol/L. WGA-Fc biding was evaluated using the same molecular proportion as the native WGA, and displayed a Kd of 6.7 × 10^−9^mol/L, which is within the same order of magnitude as the WGA (Fig. 3a). To further evaluate WGA-Fc binding to chitin, the diameters of chitin fibers were measured in the absence and in presence of the fusion protein. Chitin alone displayed populations in the ranges of 150 to 300 nm and 650 to 1300 nm (with an effective diameter of 731 nm). Addition of WGA-Fc, shifted the chitin fibers diameter ranges to 250–550 nm and 1200–2000 nm (effective diameter of 819 nm; p < 0.05), indicating aggregation of chitin fibers by WGA-Fc (Fig. 3b).

### WGA-Fc binds to *Ascomycetes* and *Basidiomycetes* fungi

**Figure 4:**
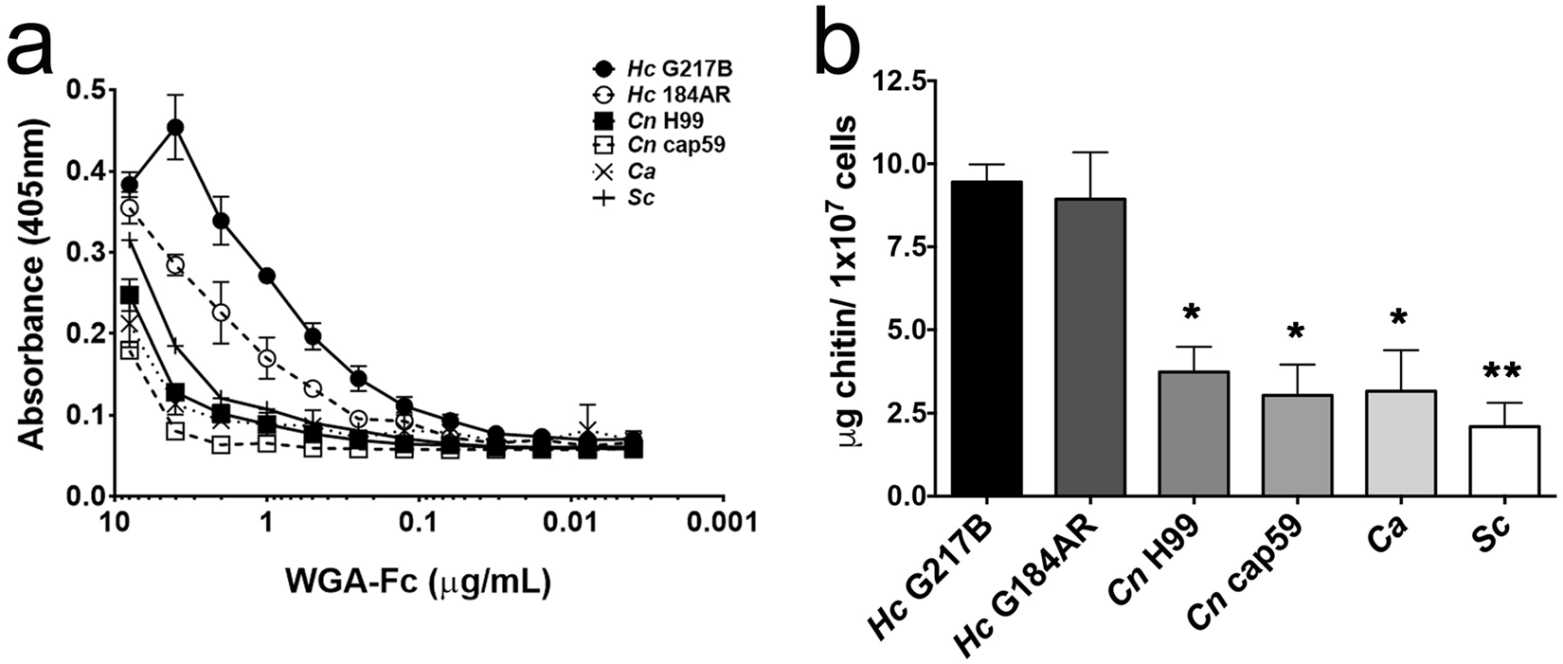
Characterization of WGA-Fc binding to pathogenic yeasts. (**a**) Indirect ELISA displaying the affinity of the chimera to *H. capsulatum* (G217B and G184AR), *C. neoformans* (capsular H99 and acapsular cap59), *C. albicans* and *S. cerevisiae*. (**b**) Inhibition ELISA depicting the amount of chitin on the surface of the different yeasts (*p < 0.05; **p < 0.01 in comparison to *H. capsulatum* strains).

*H. capsulatum* (G217B (ATCC 26032) and G184AR (ATCC 26027)), *C. neoformans* (H99 (ATCC 208821) and cap59 (B-4131)), *C. albicans* (ATCC 90028) and *S. cerevisiae* (RSY225) yeast cells were used to coat 96-well microplates and an indirect ELISA using the decreasing concentrations of the WGA-Fc chimera was performed (Fig. 4a). The WGA-Fc chimeras displayed the highest binding to *H. capsulatum*, especially to the α-1,3-glucan lacking G217B strain (p = 0.0005). The binding of WGA-Fc was similar among *S. cerevisiae*, *C. albicans*, and *C. neoformans* H99 and cap59.

Similarly, in the inhibition ELISA, in which fungal cell suspensions were incubated with WGA-Fc, unbound chimera was detected by binding to chitin oligomers. Comparison to a chitin standard curve, allowed indirect estimation of the amount of chitin on the surface of the different fungi. As with the indirect ELISA, the chitin content was higher on *H. capsulatum* (G217B and G184AR strains), followed by *C. neoformans* (H99 and cap59 strains), *C. albicans* and *S. cerevisiae* (Fig. 4b).

### WGA-Fc binding pattern to pathogenic and non-pathogenic yeasts

**Figure 5:**
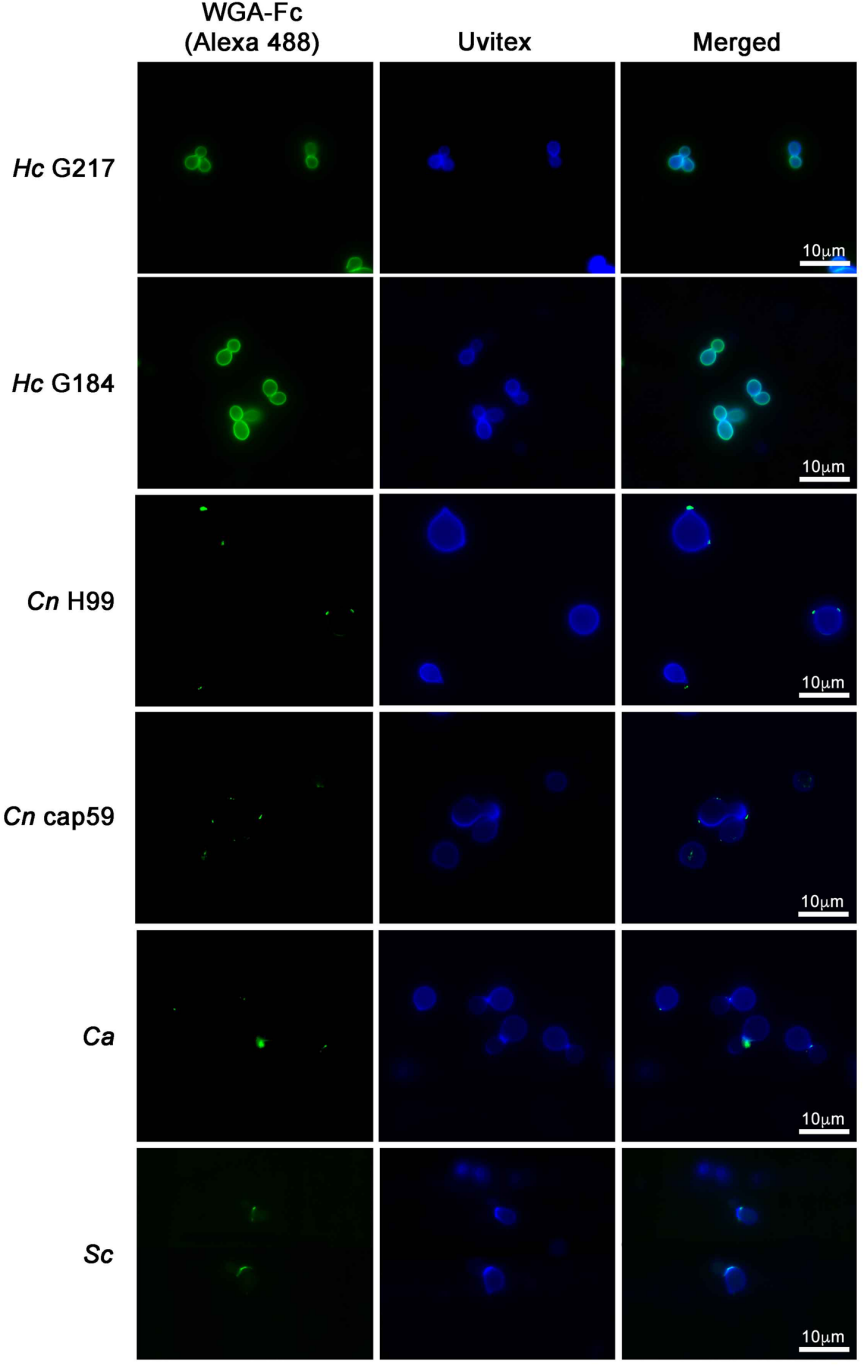
Immunofluorescence microscopy showing the labeling pattern of different fungi by WGA-Fc (Alexa 488) and Uvitex (denoting the cell wall dimensions). Binding of WGA-Fc to *H. capsulatum* yeast cells (G217B and G184AR) occurred in a homogeneous ring pattern on the cell wall, which co-localized with Uvitex (blue). WGA-Fc bound *C. neoformans* (capsular H99 and acapsular cap59) in a punctuate pattern, with binding localized to opposite poles of the cell wall, which was external to the Uvitex labeling. *C. albicans* and *S. cerevisiae* were both labeled in a punctuate fashion at the budding neck, and the binding co-localized with Uvitex staining. Scale bar = 10 μm.

The chimeric protein bound the cell walls of the different fungi (Fig. 5), albeit with differences in the labeling patterns. *H. capsulatum* strains G217B and G184AR both displayed a smooth ring pattern for the WGA-Fc binding, which co–localized with the Uvitex staining of chitin. *C. neoformans* strains H99 and cap59 displayed a similar punctate pattern of WGA-Fc binding at opposite poles on the cell, and labeling was more peripheral than that of Uvitex chitin labeling. WGA-Fc primarily labeled *C. albicans* at the bud neck between yeasts or at bud scars, and the depth of binding was similar to that of Uvitex labeling of chitin (Fig. 5). For *S. cerevisiae*, labeling by WGA-Fc was similar to that seen with *C. albicans* yeast cells, but the binding pattern was more diffuse and spread out from the zone of budding.

### WGA-Fc increased phagocytosis of pathogenic yeasts by macrophages

**Figure 6:**
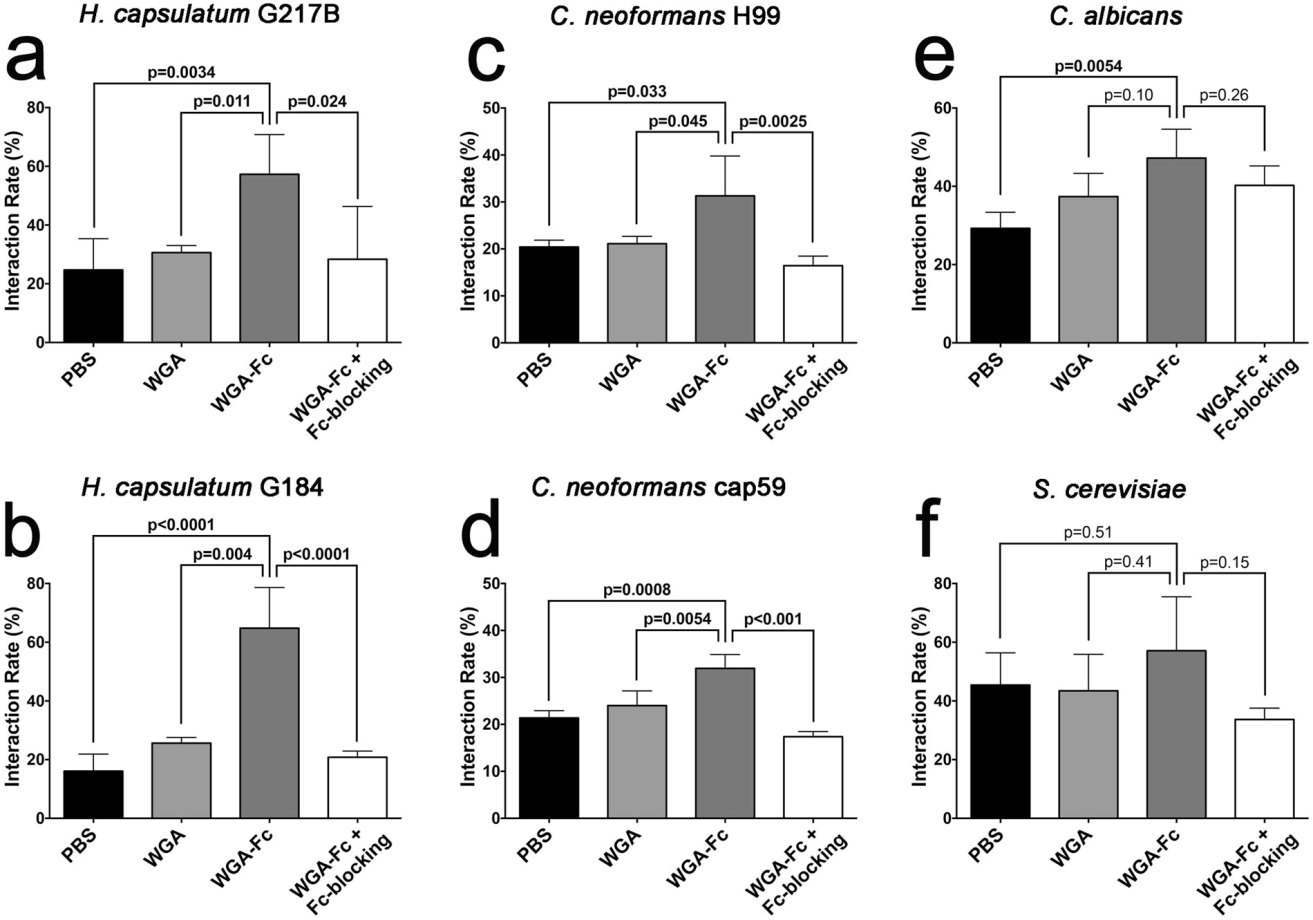
Graphical representation of interaction rates for yeast cells opsonized with PBS, WGA or WGA-Fc (and WGA-Fc/Fc blocking mAb) for 1 h prior to macrophage co-culture with distinct fungi. The interaction rate was determined by the ratio of macrophages associated with fungi over the total macrophages as determined by flow cytometry and multiplied by 100 for percentage calculations. The data shown are the averages and standard deviations of three independent experiments. For each fungus, comparison among groups was performed by One-way ANOVA, and individual comparison between control (either PBS or WGA) and WGA-Fc groups was performed using Bonferroni post-test. *p* < 0.05 was considered statistically significant.

Given the ability of the WGA-Fc to bind to the cell wall, we explored the capacity of the chimeric protein to facilitate fungal interaction with macrophages. Except for *S. cerevisiae*, pre-incubation of WGA-Fc significantly increased the association of the fungal species with macrophages compared to PBS control. Blockage of Fc receptors in the presence of WGA-Fc reduced phagocytosis to levels similar to untreated fungi or WGA controls. WGA-Fc increased the interaction of *H. capsulatum* G217B by 132%, 87% and 102% when compared to PBS, WGA and WGA-Fc/Fc-blocking, respectively (WGA-Fc 57.3% vs. PBS 24.7%, p = 0.0034, vs WGA 30.6%, p = 0.011 and vs WGA-Fc/Fc-blocking 28.4%, p = 0.024; Fig. 6a). The impact of the chimeric protein on association was even greater with *H. capsulatum* G184AR, where WGA-Fc increased the interaction by 303%, 153% and 211% when compared to PBS, WGA and WGA-Fc/Fc-blocking, respectively (WGA-Fc 64.8% vs. PBS 16.1%, p < 0.0001, vs. WGA 25.6%, p = 0.004 and vs WGA-Fc/Fc-blocking 20.8%, p < 0.0001; Fig. 6b). WGA-Fc also significantly enhanced the interaction of both *C. neoformans* H99 and cap59 compared to PBS, WGA and WGA-Fc/Fc-blocking (Fig. 6c and d, respectively). Interestingly, WGA-Fc increased the association of *C. albicans* yeast cells relative to PBS, but not WGA or WGA-Fc/Fc-blocking, albeit there was a trend toward enhancement (WGA-Fc 47.2% vs. PBS 29.3%, p = 0.0054, vs. WGA 37.4%, p = 0.10 and WGA-Fc/Fc-blocking 40.23%, p = 0.26; Fig. 6e). WGA-Fc did not significantly alter the interaction of *S. cerevisiae* with macrophages (Fig. 6f).

### Opsonization with WGA-Fc enhances the antifungal efficacy of macrophages

**Figure 7:**
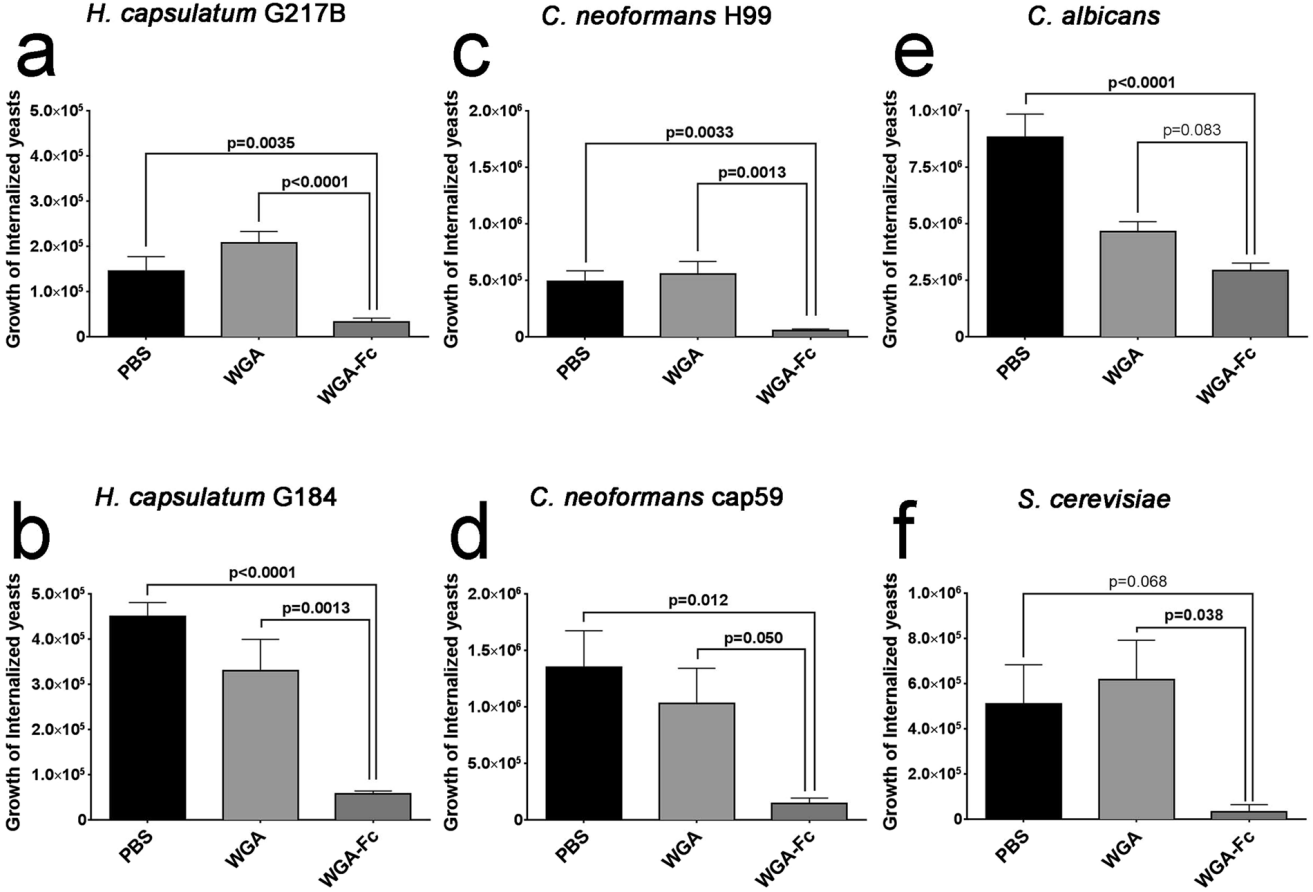
Graphical representation of fungal growth inhibition by macrophages. The ability of macrophages to restrict fungal growth is shown for yeast cells opsonized with PBS, WGA or WGA-Fc for 1 h prior macrophage co-culture, followed by washing to remove extracellular fungi and an additional 18 hour of co-culture. Growth is represented by CFU counts. Experiments were done in duplicates and the results shown represent the average at least three independent experiments. For each fungus, comparison among groups was performed by One-way ANOVA, and individual comparison between control (either PBS or WGA) and WGA-Fc groups was performed using Bonferroni post-test. *p* < 0.05 was considered statistically significant.

Opsonization of yeast cells with WGA-Fc prior to co-culture with macrophages altered the rates of intracellular fungal growth. Opsonization of *H. capsulatum* G217B yeasts prior to macrophage co-culture reduced CFUs by 77% and 84% when compared to PBS and WGA controls, respectively (WGA-Fc 3.3 × 10^4^ CFUs vs. PBS 1.4 × 10^5^, p = 0.0035 and vs. WGA 2.1 × 10^5^, p < 0.0001; Fig. 7a). WGA-Fc opsonization of *H. capsulatum* G184 similarly reduced the CFUs, by 87% and 82% when compared to PBS and WGA, respectively (WGA-Fc 5.8 × 10^4^ CFUs vs. PBS 4.5 × 10^5^, p < 0.0001 and vs. WGA 3.3 × 10^5^, p = 0.0013; Fig. 7b). Pre-incubation with WGA-Fc significantly reduced the CFUs of both *C. neoformans* H99 and cap59 by at least 86% compared to PBS or WGA (Fig. 7c and d, respectively). Pre-incubation of *C. albicans* with WGA-Fc reduced the CFUs, but significance was only achieved relative to PBS, with a reduction of 67% (WGA-Fc 2.9 × 10^6^ CFU vs. PBS 8.8 × 10^6^, p < 0.0001 and vs. WGA 4.7 × 10^6^, p = 0.083; Fig. 7e). Although there were no differences for phagocytosis, opsonization of *S. cerevisiae* induced a CFU reduction of 95% compared to PBS and WGA controls (WGA-Fc 3.3 × 10^4^ CFU vs. PBS 5.1 × 10^5^, p = 0.068 and vs. WGA 6.2 × 10^5^, p = 0.038; Fig. 7f).

### WGA-Fc directly inhibits fungal growth

**Figure 8:**
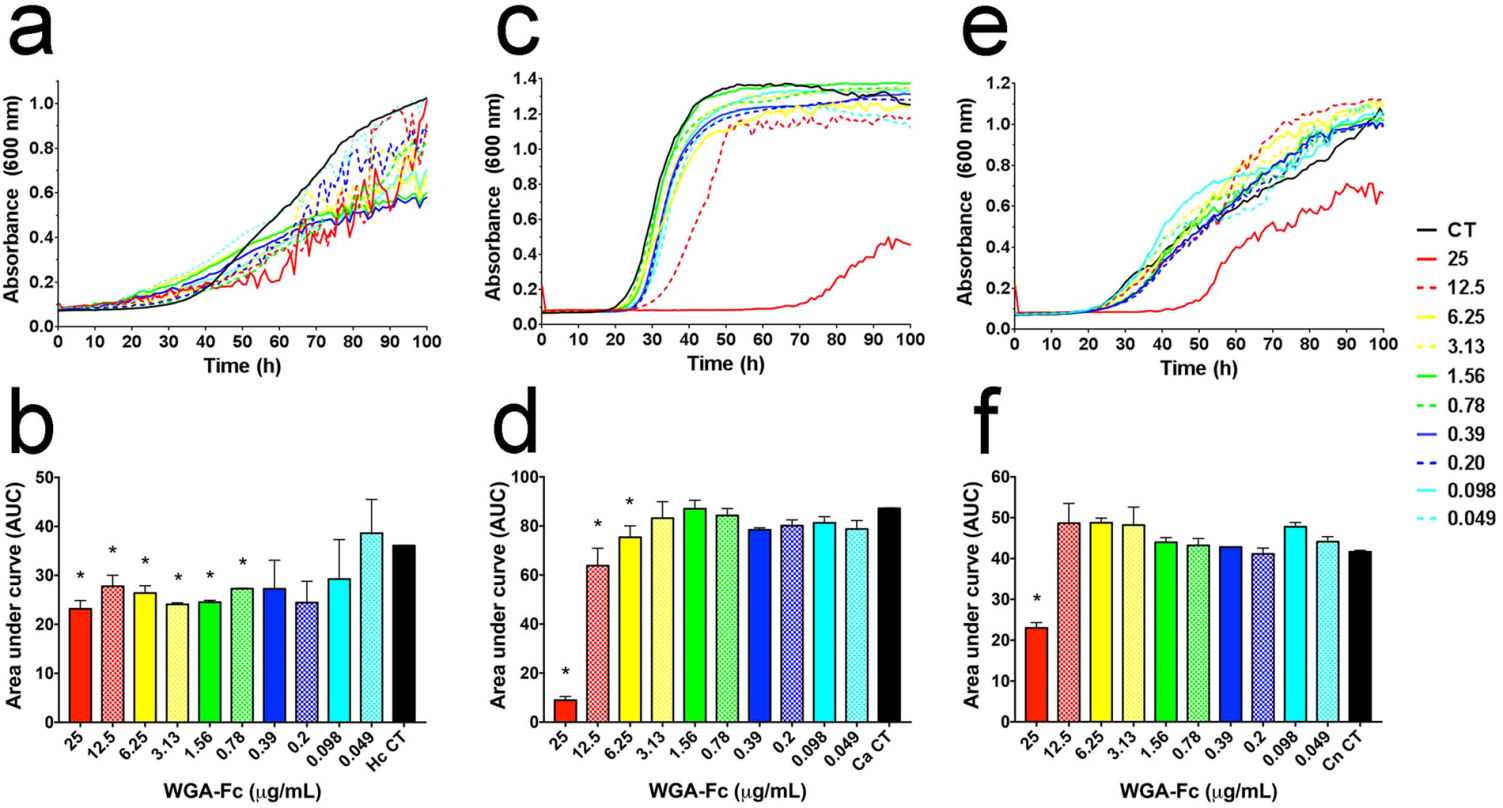
WGA-Fc altered the growth kinetics of the fungi evaluated. Bioscreen average growth curves obtained after cultivation of (**a**) *H. capsulatum*, (**c**) *C. albicans* and (**e**) *C. neoformans* at 37 °C in the presence of decreasing concentrations of WGA-Fc, ranging from 25 μg/mL to 0.049 μg/mL. The area under the growth curves of decreasing concentrations of fusion proteins WGA-Fc were calculated and individually compared to a control curve (CT) in the absence of WGA-Fc for (**b**) *H. capsulatum*, (**d**) *C. albicans* and (**f**) *C. neoformans* (*p < 0.05).

Addition of different WGA-Fc amounts (0.049 µg/mL to 25 µg/mL) to *H. capsulatum*, *C. neoformans* or *C. albicans* cultures resulted in changes of fungal growth kinetics (Fig. 8). A more prominent inhibition effect was observed with *H. capsulatum* (Fig. 8a), whose growth was inhibited at WGA-Fc concentrations as low as 0.78 µg/mL (Fig. 8b, *p < 0.05). *C. albicans* growth inhibition by WGA-Fc was also observed (Fig. 8c), with minimal inhibitory concentrations down to 6.25 µg/mL (Fig. 8d, *p < 0.05). *C. neoformans* was the least susceptible species (Fig. 8e), with only the highest concentration of 25 µg/mL causing fungal growth inhibition (Fig. 8f, *p < 0.05). As a control, equivalent minimum inhibitory concentrations of WGA provided indistinguishable results to WGA-Fc (data not shown). On the basis of these results, *H. capsulatum* and *C. albicans* were then selected for additional assays of WGA-Fc antifungal potential.

### *C. albicans* germ tube inhibition by WGA-Fc

Germ tubes of *C. albicans* were induced by the addition of serum. As described, WGA-Fc bound yeasts of *C. albicans* in a punctuated fashion, whereas binding intensified as filamentation progressed (Supplementary Fig. S1a). The addition of WGA-Fc under conditions of germ tube induction blocked filamentation in a significant percentage of the yeast population, as well as reduced the length of germ tubes (Supplementary Fig. S1b).

### WGA-Fc complement activation and *H. capsulatum* growth inhibition

The ability of WGA-Fc to activate the complement pathway was evaluated using *H. capsulatum* yeast and WGA-Fc as model, and cells were evaluated by either microscopy and flow cytometry (Supplementary Fig. S2a and S2b, respectively). Untreated or heat-inactivated serum treated *H. capsulatum* were not labeled by an anti-C3 antibody. *H. capsulatum* incubated with mouse serum displayed a discrete surface labeling for the deposition of C3 protein (Supplementary Fig. S2a). Fungal cells incubated with either WGA-Fc-added serum or from WGA-Fc intraperitoneally injected mouse serum, displayed a mix of ring and punctuated labeling patterns. Fluorescence intensities related to C3 surface deposition were also confirmed by FACS and correlated to fluorescence images (Supplementary Fig. S2b). The addition of mouse serum together with WGA-Fc reduced the growth of the fungus by 79% relative to growth in serum without WGA-Fc at 48 h (Supplementary Fig. S2c), whereas the addition of only WGA-Fc to *H. capsulatum* slowed growth by 72%.

### WGA-Fc protected mice against a lethal challenge with *H. capsulatum*

**Figure 9:**
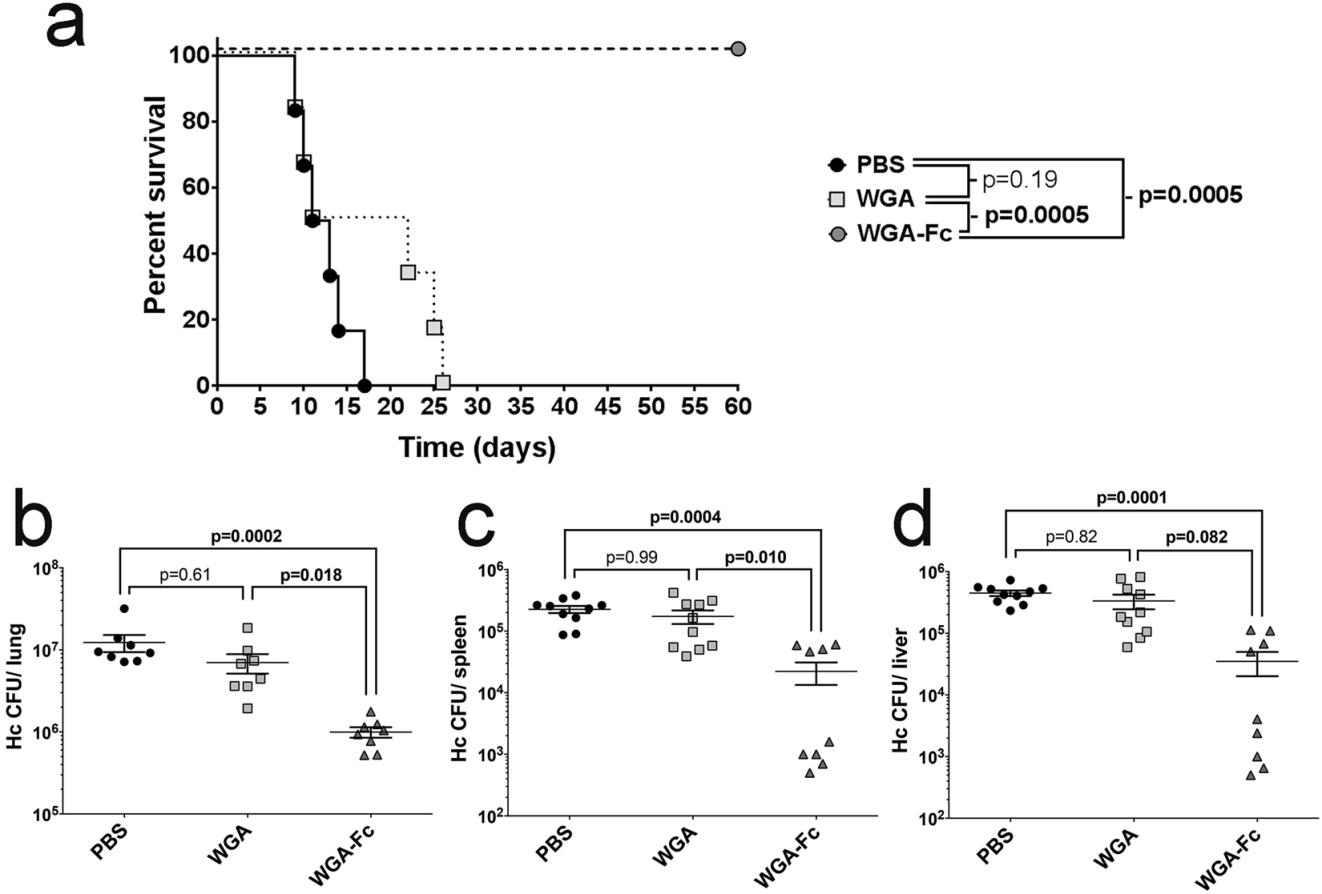
Administration of WGA-Fc is protective in a mouse *H. capsulatum* infection model. (**a**) Survival experiments comparing PBS, WGA and WGA-Fc treated groups that received a lethal challenge with *H. capsulatum*. Animals from PBS and WGA control groups died between days 9–26, whereas all WGA-Fc treated mice survived for the duration of the experiment (p = 0.0005). For the fungal burden experiments, animals were infected with a sub-lethal inoculum and treated with PBS, WGA or WGA-Fc. CFUs were determined in (**b**) lungs, (**c**) spleen and (**d**) liver. WGA-Fc treatment decreased CFUs by at least 1-log in all organs compared to PBS or WGA treated groups. The survival experiment was performed twice with similar results and the organ fungal burden data shown represents the average of two independent experiments.

Mice were lethally challenged with an intranasal inoculum of 1.25 × 10^7^ *H. capsulatum* yeast cells after administration of PBS or 10 µg of either WGA control or the WGA-Fc chimera. Animals in the PBS group died by day 17 post-infection, while the WGA treated mice died by day 26; there was no statistical difference between these control groups. In sharp contrast, all of the animals treated with WGA-Fc survived the lethal challenge through the duration of the experiment (60 days; p = 0.0005 compared to PBS or WGA control groups; Fig. 9a).

To further evaluate the protective effect of the chimeras, animals were infected with a sublethal inoculum of 5 × 10^6^ *H. capsulatum* yeast cells and treated as described. At day 7 of infection, *H. capsulatum* CFUs were determined in the lungs, spleen and liver of each mouse. Treatment with the chimeric protein significantly reduced the CFUs in the lungs compared to PBS and WGA (WGA-Fc 9.9 × 10^5^ CFUs vs. PBS 1.2 × 10^7^ CFUs, p = 0.0002 and vs. WGA 7.1 × 10^6^, p = 0.018; Fig. 9b). WGA-Fc treatment also significantly reduced the CFUs in the spleens and livers compared to either PBS or WGA (Fig. 9c and d, respectively).

### Histopathological evaluation displays lower inflammation and maintenance of tissue architecture in the lungs of WGA-Fc treated *H. capsulatum* infected mice

**Figure 10:**
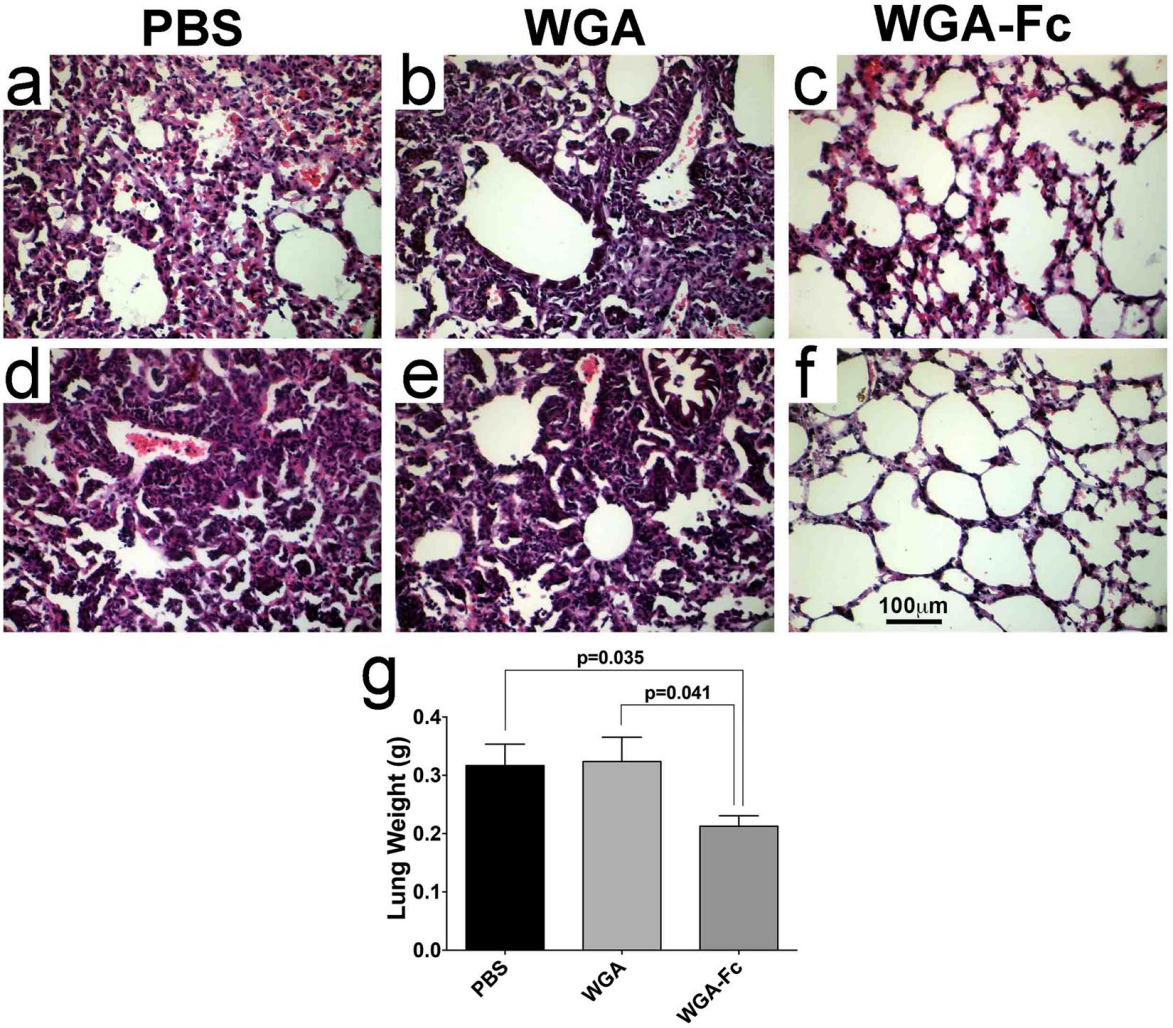
Administration of WGA-Fc protects the lung architecture of mice infected with *H. capsulatum*. Histological evaluations of lungs from mice treated with PBS, WGA or WGA-Fc and infected with a sub-lethal inoculum of *H. capsulatum*. Pictures shown are representative of lung architecture observed for each group. Central lung regions were compared for (**a**) PBS, (**b**) WGA and (**c**) WGA-Fc, where intense inflammatory changes were widely present in PBS and WGA treated mice, whereas only patchy areas of peri-bronchiolar inflammation occurred in WGA-Fc treated animals. The peripheral lung regions were also compared (**d**) PBS, (**e**) WGA and (**f**) WGA-Fc. Diffuse inflammation was present in the PBS and WGA treated mice, but the pulmonary architecture was largely normal in the WGA-Fc treated animals. Scale bar = 100 μm. (**g**) Lungs from animals of individual groups were weighted and averages of each group compared by One-way ANOVA, with individual comparisons performed using Bonferroni post-test.

Histopathological evaluations were performed concurrently with the CFU experiments. Histological examination of *H. capsulatum* infected lungs revealed structural differences among the PBS, WGA and WGA-Fc treated groups. PBS and WGA treated mice displayed a similar pattern characterized by the diffuse presence of an intense monocytic cell infiltrate as well as inflammatory and non-caseating granulomatous cell aggregates, filling most of the alveolar space (Fig. 10a and b, respectively). Areas of dense pneumonia were also observed throughout the lung (Fig. 10d and e). In marked contrast, WGA-Fc treatment significantly reduced pulmonary inflammation in response to *H. capsulatum*, as histological analysis revealed only scattered peri-bronchiolar inflammation in central regions of the lungs (Fig. 10c) with the majority of the lung displaying relatively normal alveolar airspaces (Fig. 10f). This data correlates with the lung weights of animals from the respective treatment groups, with the lungs from the WGA-Fc treated group being significantly lighter (0.21 ± 0.042 g) in comparison to the PBS (0.32 ± 0.037 g; p = 0.035) and WGA treated (0.32 ± 0.042 g; p = 0.041) groups (Fig. 10g).

### WGA-Fc administration reduced splenic burden of *C. neoformans*

We also evaluated whether treatment of lethally infected mice with WGA-Fc conferred protection against a lethal intratracheal infection with *C. neoformans*. Although there was a trend toward increased survival in WGA-Fc treated mice, the prolongation was not statistically significant (Supplementary Fig. S3a). In animals that were euthanized 7 days after challenge, treatment with WGA-Fc did not alter either pulmonary or cerebral CFUs (Supplementary Fig. S3b & S3c, respectively). However, treatment with either WGA or WGA-Fc reduced the splenic CFUs in 65.2 and 77.1% relative to control counts, respectively (Supplementary Fig. S3d).

## Discussion

New therapies are urgently required to improve the treatment of fungal infections. Over the last two decades, there has been renewed interest in immunotherapeutics, especially as these approaches have the potential to overcome antimicrobial resistance^7,29^. Several investigators in different laboratories have now convincingly demonstrated that antibodies to fungal antigens can protect against mycosis, tearing down the notion that humoral immunity is only responsible for resistance against extracellular pathogens and immunological defenses against mycosis are only cell mediated^12,13^. The majority of reports describe protective efficacy of antibodies against fungal surface antigens^15,16,19,38–40^ shared by several pathogenic fungal species. Therefore, this cross-reactivity could be leveraged to extend the action of the antibodies for the treatment of diverse fungal infections^13,41^. Thus, the application of this hypothesis could eliminate the need for specific diagnosis of fungal infection, which often requires time-consuming techniques^41^, prior to initiating treatment^42^. This approach could also reduce the use of ineffective antifungals, which in turn would diminish toxicity and shorten the therapies currently applied.

Recently, a panel of “pan-mAbs” against different *C. neoformans* and *C. albicans* cell surface antigens were conjugated to either 188-Re or 213-Bi, and these radiolabeled mAbs exhibited fungicidal activities against these yeasts^6^. Additionally, one of the mAbs produced against Hsp60 of *H. capsulatum* has been shown to have protective effects in an experimental model of *Paracoccidioides lutzii* ^43^. Hence, there is precedence for the development of “pan-fungal mAbs”.

Cell wall carbohydrates are relatively conserved among phylogenetically distant fungal species independently of cell wall organization. Hence, for the development of a “pan-fungal” therapeutic, we focused on chitin, as it is a major component of fungal cell walls and is recognized by mannose receptors on phagocytic cells. We posited that mAbs to this “common” or “universal” antigen could bind and inhibit the growth of diverse fungal species. Since the generation of a humoral response to carbohydrates is technically difficult, by demanding T-cell-independent responses and usually results in low affinity antibodies to these molecules^44^, we circumvented these challenges by generating an antibody-like fusion protein consisting of WGA, a lectin with high affinity to chitin, linked to a constant Fc-effector region. We postulated that the chimeric WGA-Fc would have the capacity to bind chitin and function as an immunoglobulin IgG2a, allowing recognition by activating FcR, triggering phagocytosis and inducing antifungal activities of the effector cells, such as the release of nitric oxide and reactive oxygen species^17,36,45^.

Biochemical characterization of the WGA-Fc chimera molecular weight by Western blot, demonstrated the binding of two monomers WGA-Fc´ through disulfide bonds, to form an antibody-like structure with two binding arms consisting of WGA lectin, linked to a structure resembling the Fc domain of an IgG2a. However, sample preparation for this evaluation could result in protein degradation into additional bands as a regular limitation of the technique. This limitation was overcome by using physiological conditions and DLS for measurement of the WGA-Fc fusion protein in solution, which had a doubled effective diameter when compared to denaturing conditions.

The comparison of the binding affinity of WGA-Fc chimeras and native WGA to chitin revealed that they had similar dissociation constants, demonstrating that the presence of the Fc region bound to WGA did not alter the WGA binding capacity to its target. Therefore, the WGA-Fc displayed distinct binding efficiency to each fungus, which could be explained by the relative amount of chitin on distinct fungal surfaces among the species studied. Neither the presence or absence of α-1,3-glucans on *H. capsulatum* strains or a capsule on *C. neoformans* altered the binding capacity of the WGA-Fc to fungal surfaces, suggesting that the WGA-Fc’s size does not influence its ability to reach the native target at the fungal cell wall despite of its complexity.

In our studies, Uvitex 2B was used to detect and delimits cell wall dimensions by total chitin staining on fungal surfaces^46^, as it displays superior results when compared to other stains such as calcofluor^47^. The lectin WGA in turn, also binds chitin, although it usually recognizes chitin oligosaccharides with greater affinity^48,49^. Then, the binding pattern of the WGA-Fc fusion protein to fungal surfaces and co-localization of WGA-Fc and Uvitex 2B was evaluated and displayed heterogeneity depending on the species. The fungus *H. capsulatum* displayed a homogeneous ring patter of WGA-Fc labeling, indicating a greater accessibility or high amounts of chitin oligomers on the cell wall of this fungus relative to the other species^50^. similar observations had been previously made in studies to characterize the *H. capsulatum* yeasts phase specific (Yps3) protein with affinity to fungal chitin. Binding of WGA-Fc to either capsular or acapsular *C. neoformans* yeasts, displayed a punctuated pattern on the cell wall, labeling the chitin oligomers at the outer level as Uvitex 2B, in accordance with previous data that described the association of chitin oligomers to yeast budding or glucuronoxylomannan anchoring sites^34^. With *C. albicans* and *S. cerevisiae*, WGA-Fc labeling concentrated on the budding neck of both yeasts, indicating chitin hydrolysis by cell wall remodeling and higher concentration of chitin oligomers on these sites, as expected according to previous findings^51,52^.

Chitin is recognized by peritoneal macrophages via TLR-2 and the mannose receptor, which can also increase phagocytosis^53^. Opsonization with WGA-Fc enhanced fungal interactions with bone marrow-derived macrophages, with the exception of *S. cerevisiae*, and these results could be correlated directly by the amount of chitin/ WGA-Fc binding on the surface of each fungus. However, for *S. cerevisiae*, despite similar interaction rates to controls, the presence of the Fc region on the WGA-Fc fusion protein should promote phagocytosis primarily through activating-Fc receptors^17,35^. Consistently, the opsonization with WGA-Fc and interaction through activating-Fc receptors mediated by WGA-Fc significantly increased the antifungal activity of these phagocytic cells and, despite the lack of enhanced interaction, *S. cerevisiae* yeasts, were nearly eradicated, underscoring the differences in pathways into the macrophages and their subsequent activation state.

Binding of WGA-Fc to fungal surfaces changed several biological aspects of the yeasts, resulting in *in vitro* growth inhibition, and the fungistatic efficiency varied in each specific model. This observation led us to explore additional mechanisms by which the chimera could exert an antifungal activity. *C. albicans* is well known by its formation of germ tubes and hyphae during infection, which is essential for pathogenesis^54,55^. The capacity of WGA-Fc to inhibit germ tubes could result in reduced host tissue invasion and biofilm formation, resulting in a protective efficacy of this molecule *in vivo*. Presumably, WGA or WGA-Fc would be expected to induce the same outcome by direct growth inhibition, as both molecules recognize the same chitin oligomers. However, in contrast to WGA alone, WGA-Fc has the potential advantage to active the complement system. For *H. capsulatum*, the presence of serum has no effect on fungal growth^56,57^. However, the presence of the WGA-Fc enhanced the complement deposition of the fungal surface, resulting in fungal death. Such *in vitro* effects suggest that the WGA-Fc has the potential for *in vivo* efficacy.

In fact, the prophylactic administration of WGA-Fc prior to a lethal challenge with *H. capsulatum* resulted in the survival of 100% of the WGA-Fc treated mice. This protective efficacy was associated with a reduction in lung, spleen and liver fungal burdens of *H. capsulatum*. Moreover, the lower lung CFUs in the WGA-Fc treated mice was accompanied by a dramatic reduction in lung injury. This reduction in inflammation was associated with lower IFN-γ and increased IL-4 and IL-10 levels (data not shown), which suggests that WGA-Fc therapy may both result in a more rapid resolution of the infection and diminished sequelae of disease^58–61^, such as complications of fibrosis^62^. Although less efficacious in a model of cryptococcosis, WGA-Fc administered prior to challenge with *C. neoformans* was able to reduce splenic fungal burdens. However, it is possible that different dosing of the WGA-Fc could enhance its efficacy or that combining the WGA-Fc with standard antifungals could produce an additive or synergistic effect.

The design of modified-mAbs to fungal antigens bearing a lectin-binding site is herewith shown to be an important tool for the generation of antibody-like reagents with high affinity to polysaccharides. The utilization of the state-of-the art techniques for the combination of distinct molecules for the construction of these types of reagents presents numerous possibilities by combining a wide variety of lectins to distinct Fc regions of mAb genes. Also, lectin-Fc against these “common” targets could readily be used against phylogenetically distant fungi, since these antigens are conserved among different species. We propose that these antibody-like chimeras should be considered as “universal” instead of “cross reactive”, as the latter term is largely used to represent a lack of diagnostic specificity.

Overall, our data suggests that as a therapeutic, the WGA-Fc may be a potentially powerful prophylactic in patients at high risk for invasive mycoses as well as a biologic for use in the treatment of active disease. Future translational studies, including in immunocompromised animal infection models, are needed to advance this “universal” chimeric protein into clinical trials.

## Methods

### Animal use and ethics statement

All animal experiments were performed under approved protocols by the Institute for Animal Studies of the Fluminense Federal University, NAL (Núcleo de Animais de Laboratório), Protocol Number NAL 600, under guidelines established by Conselho Nacional de Controle de Experimentação Animal (CONCEA, Law number 11.794/2008 and Resolution number 3, December/2011).

### Organisms and growth conditions

The hybridoma cell line 15.5.5S expressing an IgG2a was obtained from the Cell Bank of Rio de Janeiro and cultured in DMEM medium (ThermoFisher Scientific) supplemented with 10% fetal calf serum (Cultilab, Brazil), 1% non-essential amino acids (ThermoFisher Scientific) and 1% penicillin/streptomycin (HyClone). Chinese hamster ovary (CHO-k1) cells were a gift from Dr. Joshua D. Nosanchuk, and were cultured in HAM F-12 medium supplemented with 1.2 g/L NaHCO_3_, 5% FCS, 2% non-essential amino acids and 1% penicillin/streptomycin. CHO-k1 cell cultures were incubated at 37 °C/5%CO_2_. Two strains of *H. capsulatum*, G184AR (ATCC 26027) and G217B (ATCC 26032; lacking α-1,3-glucan on the yeast cell surface), were grown in Ham’s F-12 medium supplemented with glucose (16 g/L), glutamic acid (1 g/L), HEPES (6 g/L) and cysteine (8.4 mg/mL) at 37 °C^25^. An encapsulated *C. neoformans* strain H99 (ATCC 208821) and an acapsular mutant cap59 (B-4131) were cultivated in a chemically defined minimum medium at 37 °C^63^. *C. albicans* (ATCC 90028) and *Saccharomyces cerevisiae* RSY225 were cultivated in Sabouraud at 37 °C. *C. albicans* filamentation was induced as described previously^64^.

### WGA and Fc amplification

RNA was extracted from wheat (*Triticum aestivum*, collection of Empresa Brasileira de Pesquisa Agropecuaria- EMBRAPA) and the IgG2a producing hybridoma. cDNAs were obtained using the SuperScript III First-Strand Synthesis System according to the manufacturer’s instructions (ThermoFisher Scientific) and each cDNA was individually used as a template. The cDNA encoding the isolectin region of WGA 1 (isolectin A, GenBank AAA34256.1) was amplified by PCR with the oligonucleotide primers ggcccagccgggccCAGAGGTGCGGCGAGC (corresponding to a *SfiI* site and the 5′ end of the WGA isolectin domain) and ggatccacgcggaaccagTTCTTGGAGAAGAGTGGAGTT (corresponding to the hexapeptide linker with thrombin cleavage site and 3′ end of the WGA isolectin domain), in order to amplify the coding sequence of the isolectin domain present in WGA 1 lectin. The product was sequenced to confirm that it had 100% similarity to WGA 1 sequence (data not shown). The cDNA encoding CH2-CH3 (GenBank AB097847.1) was amplified with the oligonucleotide primers ctggttccgcgtggatccAAAATTGAGCCCAGAGGG (corresponding to the hexapeptide linker with thrombin cleavage site and 5′ end) and gaattcTCATTTACCCGGAGTCCG (corresponding to an *EcoR*I site, a stop codon and the 3′ end), in order to amplify the coding sequence of the murine IgG2a hinge and CH2-CH3 domains. The PCR reaction consisted of 100 ng of cDNA, 0.2 µM of each primer, 20 mM Tris-HCl pH 8.4, 50 mM KCl, 1.5 mM of MgCl_2_, 0.2 mM of each dNTP) and 0.25 U of Platinum DNA Taq polymerase (ThermoFisher Scientific) in 25 μL reaction. The PCR protocol followed a denaturing step of 94 °C (5 min), 35 cycles of 94 °C (1 min)/ 56 °C (1 min)/72 °C (1 min) and an elongation step of 72 °C (5 min) and was carried out in a Veriti Thermo Cycler (Applied Biosystems).

### Overlapping PCR and WGA-Fc construction

PCR products were resolved on a 1% agarose gel (Invitrogen) and purified via extraction using NucleoSpin Gel and PCR Clean-Up kit (Macherey-Nagel). The WGA and Fc fragments were combined at a 1:1 ratio and subjected to 20 cycles of denaturation and reannealing (94 °C/60 °C) to promote recombination of homologous thrombin site regions. The 5′ primer for WGA and the 3′ primer for IgG2a were used to amplify the recombinant product WGA-Fc by PCR using the same conditions as described above and product evaluated by electrophoresis on a 1% agarose gel. The chimeric PCR product was isolated and purified via gel extraction and subcloned into the TOPO Vector (ThermoFisher Scientific), which was confirmed by PCR as above. WGA-Fc was digested with *Sfi*I and *EcoR*I restriction enzymes (ThermoFisher Scientific) and inserted into the multiple cloning site of pSecTag2A expression vector (ThermoFisher Scientific), containing the IgK leader sequence facilitating protein secretion. Plasmids were sequenced to confirm the construct fidelity.

### Transfection of WGA-Fc plasmid and chimera production

For WGA-Fc production, initially CHO-k1 cells cultured in Ham’s F-12 supplemented with 10% FBS, 2% non-essential amino acids and 1% penicillin/streptomycin. Cells were transfected with 500 ng of the constructed plasmid using Lipofectamine 2000 reagent (ThermoFisher Scientific), plated on a 24-wells plate and incubated overnight as described^65^. Then, the drug zeocin (ThermoFisher Scientific) was added to the medium at 700 µg/mL for selection of plasmid harboring clones. Cells were cultured for 7 days and the supernatants of the stable clones were collected. Protein expression was detected by ELISA using chitin from shrimp shells (C9213, Sigma-Aldrich), prepared as described previously^66^. Briefly, 0.5 µg of chitin in 50 µL of PBS were added per well of a 96-wells microplate and incubated at 37 °C for 1 h, followed by an overnight incubation at 4 °C. Plates were washed and blocked with an 1% bovine serum albumin diluted in TBS-T (Tris-HCl 20 mM, NaCl 150 mM, 0.1% Tween 20, pH 7.2). Plates were washed three times with TBS and supernatants from CHO-k1 transfected cells added. After 1 h incubation, plates were washed and 50 µL of an anti-mouse IgG alkaline phosphatase conjugate at 1 µg/mL was added per well and plates incubated at 37 °C for 1 h. After washes, plates were developed with a 1 mg/mL pNPP substrate solution (Sigma-Aldrich), incubated at RT for 30 min and read in a SpectraMax microplate reader (Molecular Devices) at 405 nm.

### Cell cloning and purification of WGA-Fc chimera

The microplate well with the highest production of chimeras was chosen and the cell number expanded. Limiting dilution cloning was performed and supernatants from the new wells were re-tested for chimera production. Highest producer was expanded and its culture supernatant centrifuged for 10 min at 10000×*g* to remove cellular debris. Then, the supernatant was passed over a 1 mL HiTrap Protein A HP (GE Healthcare Life Sciences) column after equilibration with PBS (16 g/L NaCl, 0.4 g/L KCl, 0.4 g/L KH_2_PO_4_, 2.4 g/L Na_2_HPO_4_, pH 7.2). The column was washed with PBS, the bound chimera eluted with 0.1 M glycine buffer, pH 2.7 and neutralized using 1 M Tris-HCl (pH 8.0). The chimeric protein concentration was determined by inhibition ELISA as described^16^.

### Molecular characterization of the WGA-Fc chimera

To evaluate protein structure, aliquots of WGA-Fc were diluted in denaturing (12.5 mM Tris pH 6.8, 4% glycerol, 0.4% SDS, 1% ß-mercaptoethanol, 0.005% w/v bromophenol blue, dH_2_O) and non-denaturing (as above, but without 1% ß-mercaptoethanol) buffers and incubated at 95 °C for 5 min. An IgG2a mAb 12D3 against *H. capsulatum* heat-shock protein 60 KDa was used as a control^16^. The proteins were resolved by SDS-PAGE, transferred to nitrocellulose membrane and blocked with 5% milk solution in 0.1% TBS-T overnight. For immunodetection of chimeras or control antibodies, a goat anti-mouse IgG conjugated with alkaline–phosphatase was used and after washes with TBS-T, the membrane was developed with 5-bromo-4chloro-3-indolyl phosphate/NBT reagent (Sigma-Aldrich).

### Effective diameter of chimeric protein by Dynamic Light Scattering (DLS)

Size distribution of WGA-Fc was determined by Quasi-Elastic Light Scattering in a 90Plus/BI-MAS Multi Angle Particle Sizing analyzer (Brookhaven Instruments, Holtsville, NY). Chimeric WGA-Fc solutions were all used at 25 µg/ml under non-denaturing (PBS) and denaturing conditions (PBS+ 1% β-ME) and incubated at 95 °C for 5 min. Multimodal size distribution analysis of proteins was calculated from the values of intensity weighted sizes obtained from the non-negatively constrained least squared (NNLS) algorithm. Reported results are the averages of 10 different measurements.

### Evaluation of the WGA-Fc binding to chitin

The dissociation constants (Kd) of the chimeric protein and native WGA were determined by an inhibition ELISA. Chitin (50 µL of a 10 µg/mL solution in PBS) was affixed overnight to wells of a 96-well microplate and blocked with blocking solution (1% BSA in TBS-T). A second (inhibition) plate was blocked for 1 h at 37 °C, and a solution of 2 µg/mL of the WGA-Fc protein was incubated with serial 1:2 dilutions of chitin (starting at 10 µg/mL) for 1 h at 37 °C. After the incubation, the contents of the second plate were transferred to the first plate with chitin adherent/blocking. After incubation at 37 °C for 1 h, the plates were washed, and an anti-mouse IgG2a alkaline phosphatase conjugated for WGA-Fc wells and streptavidin-alkaline phosphatase conjugate for WGA-biotin were added and incubated for 1 h at 37 °C. The plates were washed again, and incubated with 1 mg/mL pNPP substrate solution (Sigma-Aldrich) at RT for 30 min. Plates were read in a SpectraMax microplate reader (Molecular Devices) at 405 nm. The Kd value was considered the chitin concentration required for 50% competition. In parallel, chitin was measured by DLS as described above in the presence and absence of WGA-Fc (0.5 µg/mL) and aggregation of chitin particles evaluated.

### Binding of WGA-Fc the pathogenic fungi used in this study

WGA-Fc chimera binding to pathogenic fungi was evaluated initially by indirect ELISA. Briefly, 50 µL of each yeast suspension (2 × 10^6^ yeast/mL) was used to coat the wells of a 96-wells microplate and incubated overnight at 4 °C. Plates were washed with TBS-T and blocked for 1 h at 37 °C. After three washes, each yeast was incubated with a serial dilution (1:2) of the WGA-Fc chimera, starting at 8 µg/mL. Plates were incubated at 37 °C for 1 h, washed three times with TBS-T and an anti-mouse IgG2a alkaline phosphatase conjugated (1 µg/mL) was added. After incubation for 1 h at 37 °C, plates were washed again, and incubated with 1 mg/mL pNPP substrate solution (Sigma-Aldrich) at RT for 30 min. Plates were read as described. Additionally, a similar inhibition ELISA was performed according to previously described protocols^67^. Briefly, serial dilutions of each fungi (starting at 10^7^ yeast cells) or a standard chitin curve (starting at 500 µg/mL) were incubated with 2 µg/mL of WGA-Fc for 1 h at 37 °C. The plate contents were transferred to a reaction plate previously coated with 50 µL of a 10 µg/mL chitin suspension. Procedures were performed as described above and fungal chitin content was extrapolated from the chitin standard curve.

### Immunofluorescence

The reactivity of the chimeric WGA-Fc was also evaluated by immunofluorescence. Briefly, in order to visualize the chimeric protein binding, 5 × 10^6^ yeast cells of each of the different fungal isolates or induced germ tubes of *C. albicans* were incubated with 1 µg/mL of WGA-Fc at RT for 1 h, followed by incubation with 1 µg/ml of goat anti-mouse IgG-Alexa 488 (Molecular Probes – Life Technologies) for 1 h at RT. Chitin in the cell wall was visualized using a 5 mg/mL solution of Uvitex 2B in PBS incubated with the yeast cells for 30 min. Cells were washed 3 times with PBS between the incubations. The samples were examined on an Axio Imager Microscope (Carl Zeiss MicroImaging, Inc.) using a 100× objective. Images were analyzed by Image J (NIH, Bethesda) and Adobe Photoshop C5S (Adobe Systems Software).

### Phagocytosis assay

Bone-marrow derived macrophage were obtained from mice as described^68^ and cultured using DMEM supplemented with 10% of FBS, 1% of streptomycin by adding approximately 4 × 10^5^ macrophages to each well of a 24-well cell culture plate, which were incubated overnight at 37 °C in 5% CO_2_. Yeast cells were washed 3 times with PBS and labeled with NHS-rhodamine (ThermoScientific) at a concentration of 40 µg/mL for 30 min at RT and then washed again 5 times with PBS. Yeasts were incubated for 1 h at RT with either 1 µg/mL of the chimeric protein WGA-Fc, 1 μg/ml WGA or PBS. After the incubation the cells were washed 3 times with PBS, and then added to macrophages (1:5 macrophage-yeast) for an 1 h incubation at 37 °C in 5% CO_2_. Wells were washed 3 times with PBS, macrophages detached and phagocytosis was arrested by adding 4% paraformaldehyde. The phagocytosis rate was determined by flow cytometry, where the percentage of macrophages interacting with fungi (FL2+ ) was determined^15,16,63^. Experiments were performed three times. Additional phagocytosis experiments were performed using macrophages pre-incubated with anti-mouse Fc receptor antibodies (rat anti-mouse CD16/32, Southern Biotechnology).

### Macrophage growth inhibition assay

The ability of macrophages to impede the growth of the fungi was evaluated by a CFU inhibition assay as described^15,16,63^. Approximately 10^5^ bone-marrow derived macrophages/well were plated in a 96-well plate using DMEM supplemented with 10% of FBS, 1% of nonessential amino acids and 1% penicillin/streptomycin and incubated overnight at 37 °C in 5% CO_2_. Yeast cultures were washed 3 times with PBS and incubated for 1 h at RT with either 1 µg/mL of the chimeric protein WGA-Fc, 1 µg/mL WGA or PBS. After incubation, yeast cells were washed 3 times with PBS, then added to macrophage cultures (1:5 macrophage-yeast) and incubated for 18 h at 37 °C in 5% CO_2_. Macrophages were lysed by adding sterile distilled water. *H. capsulatum* yeast were plated on BHI-blood agar and incubated at 37 °C for 2 weeks. Aliquots of the other fungi, *C. neoformans, C. albicans* and *S. cerevisiae* were plated on Sabouraud agar and incubated for 2 days at 37 °C. The killing activity of the chimera for each fungal isolate was expressed as absolute numbers of colony forming units (CFU) and the values were compared among the different groups.

### Assay for chimera WGA-Fc fungal growth inhibition

Growth inhibition assays were performed using broth microdilution in accordance to M27-A3 protocol of the Clinical and Laboratory Standards Institute, with some slight modifications^69^. Two-hundred µL of a 5 × 10^5^ *H. capsulatum* yeast/mL in Ham-F12, or 200 µL of a 2 × 10^3^ *C. neoformans*/mL or 10^3^ *C. albicans*/mL in RPMI 1640 (2% glucose) were incubated with concentrations ranging from 25 to 0.049 µg/mL of WGA-Fc or in the absence of this reagent for control growth curves. Plates were incubated at 37 °C for approximately 4 days in a Bioscreen (LabSystems Oy), with 600 nm absorbance readings recorded every hour, and values plotted against time to obtain the growth curves for each individual condition. The area under curve (AUC) was calculated as an integrative parameter of total growth as described elsewhere^70–72^. Values obtained in the presence of the WGA-Fc were individually compared to a control curve in the absence of the fusion protein.

### *C. albicans* germ tube formation assay

Germ tube formation by *C. albicans* was induced as described^64^, with some slight modifications. Approximately 10^5^ *C. albicans* yeasts were placed in DMEM medium supplemented with 10% FCS and added of a 10 µg/mL solution of WGA-Fc or the control. Plates were incubated at 37 °C and every 30 min, images were recorded in an Axio Imager Microscope (Carl Zeiss MicroImaging, Inc.) using a 100× objective. Images were evaluated using Image J, and the percentage of formed germ tubes and their sizes were determined at each time point and data were compared between groups.

### WGA-Fc complement activation and *H. capsulatum* growth

To evaluate the complement fixation activity of the WGA-Fc, 10^6^ yeasts of *H. capsulatum* were incubated with 5% of mouse serum (or heat inactivated mouse serum) and 2 µg/mL of WGA-Fc or individual controls for 1 h at RT. Yeasts were washed three times with PBS and incubated with a 5 µg/mL FITC-conjugated goat anti-mouse C3 (MP Biomedicals). The slides were examined as described above. For growth inhibition activity of the chimeras and complement, *H. capsulatum* was grown in the presence of WGA-Fc and mouse serum (or individual controls) and, the contents of the wells were plated after 48 h on brain heart infusion (BHI) agar supplemented with 5% (v/v) sheep blood. Plates were incubated at 37 °C for about 2 weeks and CFUs from each growth condition enumerated.

### *In vivo* models for evaluation of WGA-Fc protective efficacy

For survival experiments, C57BL/6 mice (6–8 weeks old) obtained from the Institute for Animal Studies (NAL) of the Fluminense Federal University were challenged intranasally with a lethal inoculum of 1.25 × 10^7^ *H. capsulatum* G217B or intratracheally with 10^6^ *C. neoformans* H99, 2 h after they received either 10 µg of WGA-Fc, 10 µg WGA or PBS as described previously^16,73^. Infected mice were checked four times daily by the scientific team and daily by the veterinary staff. Moribund animals were euthanized and deceased animals were enumerated.

To determine fungal burdens and assess pathological alterations, animals were challenged intranasally with a sublethal inoculum of 5 × 10^6^ *H. capsulatum* G217B or intratracheally with 10^6^ *C. neoformans* H99, 2 h after they received either 10 µg of WGA-Fc, 10 µg WGA or PBS. Mice were euthanized at day 7 post-infection and organs were removed. The left upper lobe of the lung was fixed with formalin and processed for hematoxylin-eosin staining for histological examination. The remaining lungs, as well as the spleen and liver of each mouse were weighed and homogenized in PBS using 70 µm cell strainers (BD Biosciences). For *C. neoformans*, lungs, spleen and brains of infected animals were processed. After dilutions, homogenates were plated on brain heart infusion (BHI) agar supplemented with 5% sheep blood for *H. capsulatum* determinations or Sabouraud agar plates for *C. neoformans*. BHI plates were incubated for 10–15 days and Sabouraud plates for 2 days at 37 °C. CFUs were enumerated and compared among controls and WGA-Fc treated groups for each fungus.

### Statistical analysis

All statistical analyses were carried out using GraphPad Prism version 6.00 for Windows (GraphPad Software, San Diego California USA). One-way ANOVA using a Kruskall-Wallis test was used to compare the differences among groups, with a confidence interval of 95% for all experiments. Bonferroni post-test was used for individual comparison between groups. For survival experiments, differences among groups were analyzed by Kaplan-Meier estimator.

## Electronic supplementary material

Supplementary Figures
