# Supplementary material for: Characterization of the antifungal functions of a WGA-Fc (IgG2a) fusion protein binding to cell wall chitin oligomers
Source: Sci Rep. 2017 Sep 22;7:12187. doi: 10.1038/s41598-017-12540-y (PMC5610272; doi:10.1038/s41598-017-12540-y)
Supplement: Supplementary file 1 — Supplementary Figures [file 41598_2017_12540_MOESM1_ESM.pdf]

Characterization of the antifungal functions of a WGA-Fc (IgG2a) fusion protein binding to cell wall chitin oligomers

Susie Coutinho Liedke, Daniel Zamith Miranda, Kamilla Xavier Gomes, Jorge Luis S. Gonçalves, Susana Frases, Joshua D. Nosanchuk, Marcio L. Rodrigues, Leonardo Nimrichter, José Mauro Peralta, Allan J. Guimarães

SUPPLEMENTARY FIGURE LEGENDS

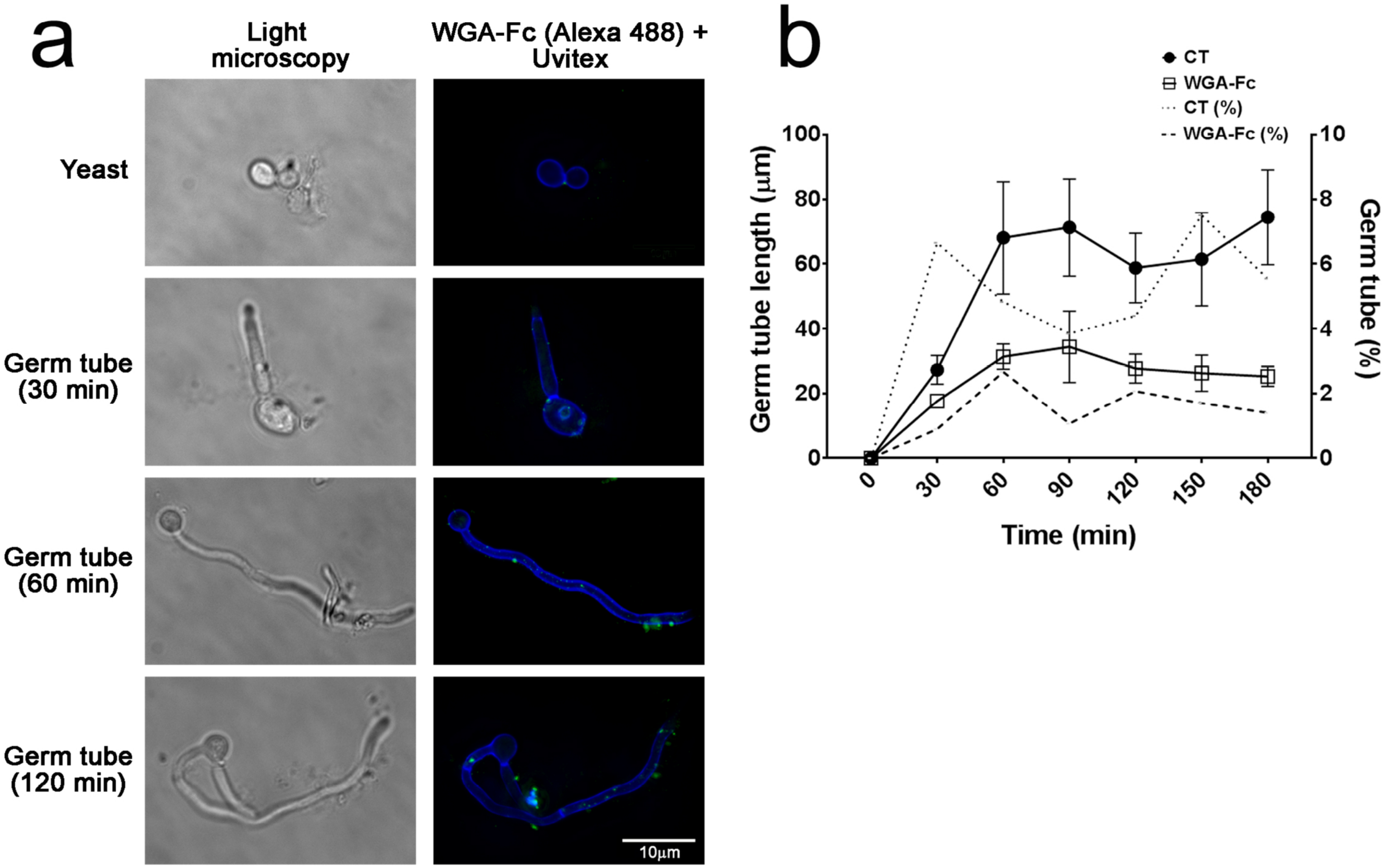

Supplementary Figure 1. WGA-Fc binds to and is able to inhibit germ tube formation of *C. albicans*. (a) Immunofluorescence microscopy showing the labeling pattern of *C. albicans* yeasts and germinative tubes by WGA-Fc (Alexa 488) and Uvitex. *C. albicans* germinative tubes were induced by serum and immunofluorescence was performed at the intervals indicated. Binding of WGA-Fc to germinative tubes was more intense when compared to yeasts, with more punctuated labeling. Scale bar = 10 μm. (b) WGA-Fc reduced both the number of *C. albicans* germ tube events and the lengths of these structures when present.

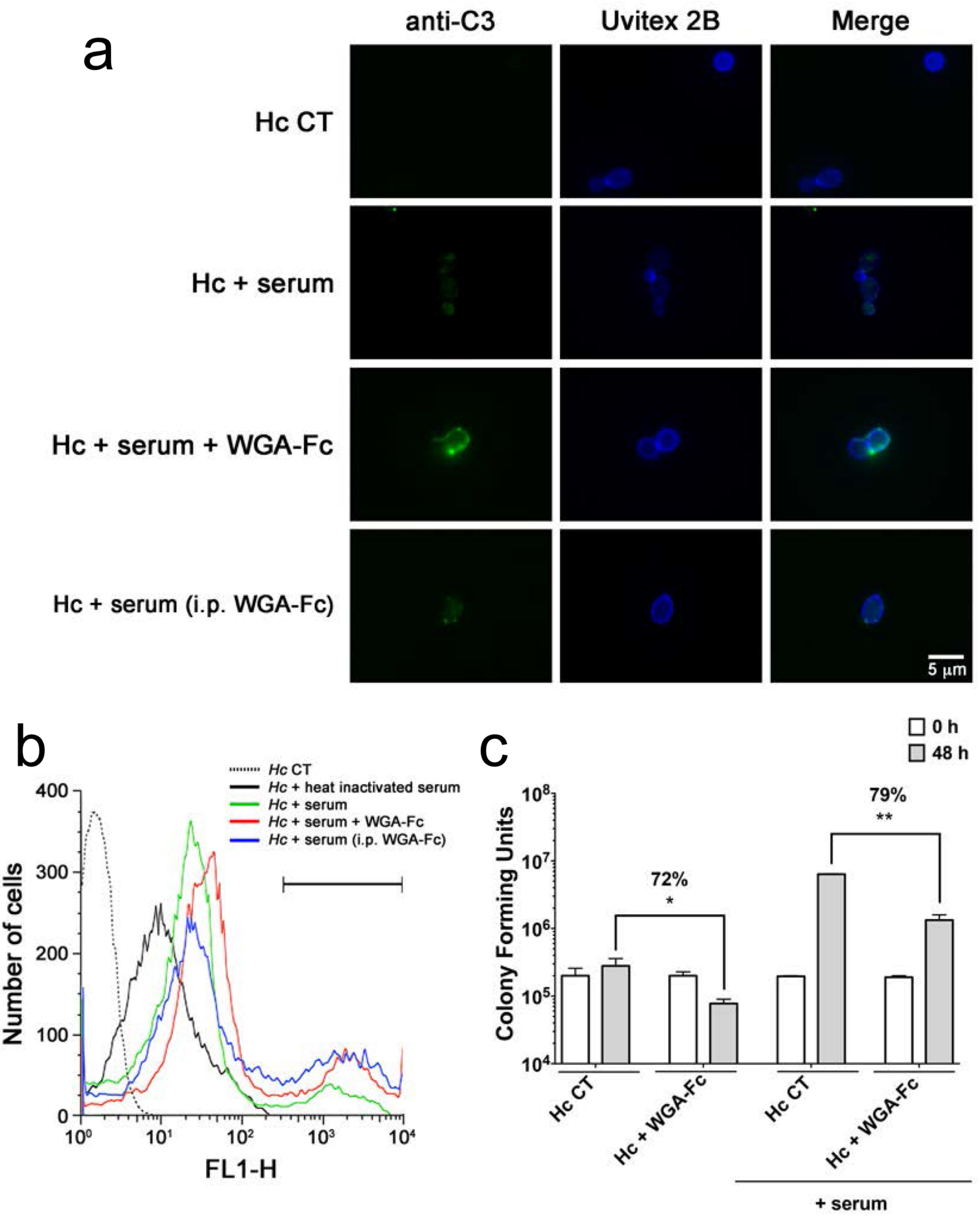

Supplementary Figure 2. WGA-Fc is able to activate the complement system, which enhanced the growth inhibition of *H. capsulatum*. (a) Deposition of serum complement proteins (C3, green fluorescence) on the surface of *H. capsulatum* is enhanced in the presence of WGA-Fc. Scale bar = 5 μm (b) Flow cytometry displaying the increase on fluorescence intensity/C3 deposition in the presence of WGA-Fc and serum in comparison to controls (Hc CT - untreated *H. capsulatum* yeasts, Hc + serum (i.p WGA-FC) – serum from animal administered intraperitoneally with WGA-Fc as described). (c) WGA-Fc displayed a fungistatic activity against *H. capsulatum* at 48 h (*$p<0.05$) which was enhanced by WGA-Fc complement activation (**$p<0.01$).

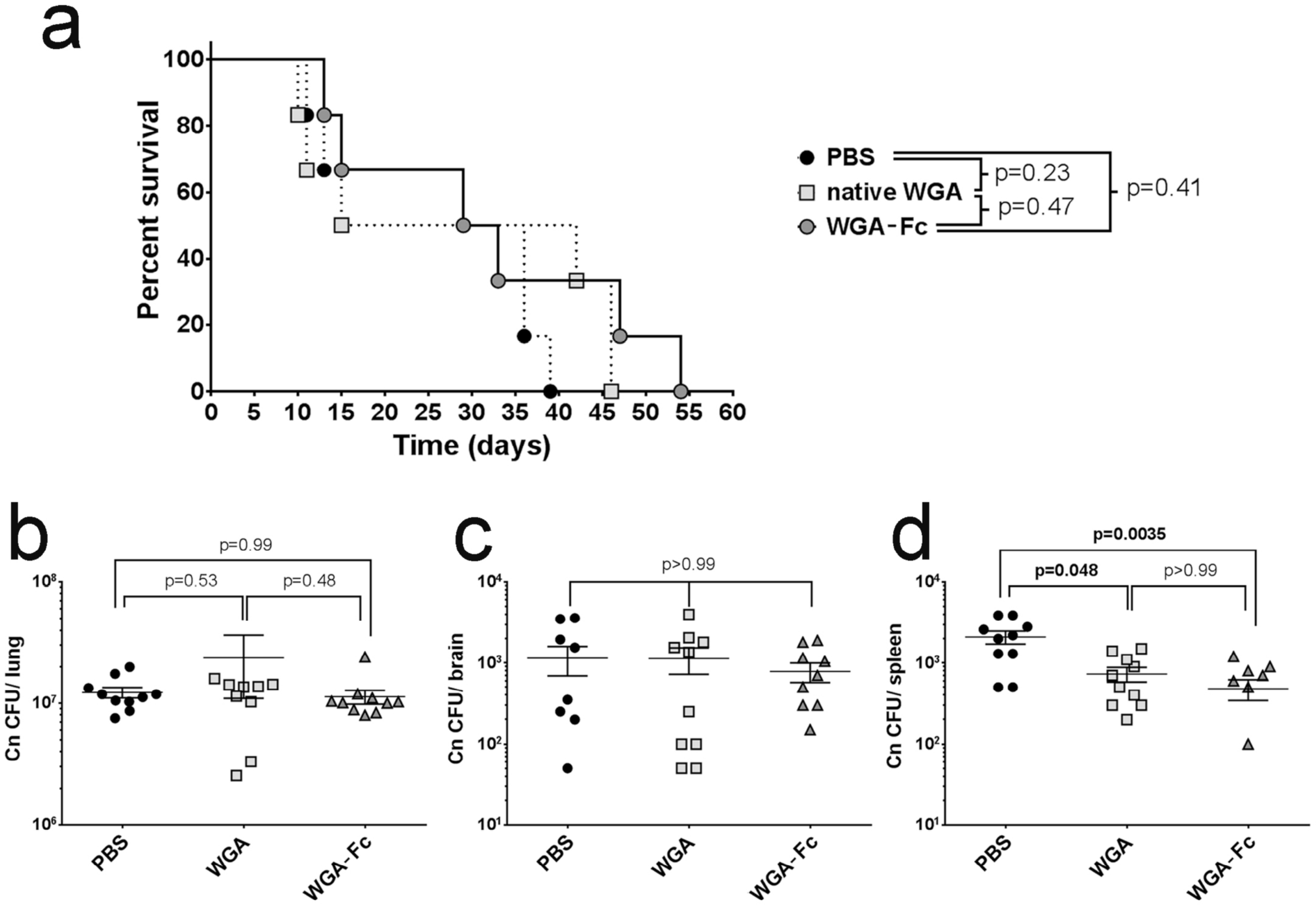

Supplementary Figure 3. Passive immunization with WGA-Fc reduced the fungal burdens of *C. neoformans* in mouse spleens. (A) Survival experiments comparing PBS, WGA and WGA-Fc treated groups that received a lethal inoculum with *C. neoformans* revealed a trend toward prolonged survival in the WGA-Fc treated mice, but no statistical difference was observed among groups. CFUs were determined in (B) lungs, (C) brain and (D) spleen. WGA-Fc treatment decreased CFUs in the spleens when compared to PBS or WGA treated groups. The survival experiment was performed twice with similar results and the organ fungal burden data shown represents the average of two independent experiments.
